# RegIIIβ promotes *Salmonella* Typhimurium colonization of the gut in the early-stage gastrointestinal infection by enhancing flagella-driven locomotion

**DOI:** 10.1371/journal.ppat.1013665

**Published:** 2025-11-03

**Authors:** Tsuyoshi Miki, Hana Yamaguchi, Momoko Kobayashi, Masahiro Ito, Takeshi Haneda, Nobuhiko Okada, Yun-Gi Kim

**Affiliations:** Department of Microbiology, School of Pharmacy, Kitasato University, Tokyo, Japan; Ludwig-Maximilians-Universitat Munchen, GERMANY

## Abstract

The bactericidal lectin RegIIIβ secreted by intestinal epithelial cells, kills the microbiota and enteropathogens but also been suggested to modulate bacterial physiology and host interactions. However, it remains to be determined whether RegIIIβ plays roles beyond its bactericidal effects. The present study revealed that RegIIIβ binds to the enteropathogen *Salmonella enterica* serovar Typhimurium in the gut, thereby increasing the locomotion speed of this bacterium through maintenance of the membrane potential. This led to enhanced invasion by *S*. Typhimurium into epithelial cells. Finally, RegIIIβ facilitated the gut colonization levels of *S*. Typhimurium and enteropathy in the early stages of gastrointestinal infection. In conclusion, *S*. Typhimurium has become tolerant to RegIIIβ in the evolutionary process, during which the pathogen has simultaneously acquired the ability to exploit this antimicrobial to enhance flagellar-based locomotion for successful gastrointestinal infection. Our findings provide novel insight into the roles of RegIIIβ in gastrointestinal infection caused by *S*. Typhimurium.

## Author summary

Foodborne diseases caused by contamination of food and water with enteropathogens, including *Salmonella*, can be severe, especially for young children and patients with weakened immunity. Thus, *Salmonella* infection accompanied by diarrhea is a major global public health concern; however, the prevention and regulation of *Salmonella* infections have had limited success. In contrast, we have evolved immune-defense mechanisms that protect us from such infections by producing antimicrobial proteins such as defensins and C-type lectins of the RegIII family, which can kill or inactivate enteropathogens. This study aims to explore the role of RegIIIβ, a C-type lectin, in *Salmonella* infections. Our findings reveal the unexpected role of RegIIIβ in enhancing the infectivity of *Salmonella*. Despite its known bactericidal properties, RegIIIβ does not kill *Salmonella* but instead binds to it, promoting its motility and colonization in the gut. These findings challenge the traditional understanding of immune defense mechanisms and open new avenues for developing strategies to combat *Salmonella* infections.

## Introduction

The gastrointestinal tract plays a protective role by providing a barrier against enteropathogens in the lumen. This barrier employs multiple innate immune systems to prevent colonization by enteropathogens and eliminate them from the intestinal tract. Antimicrobial proteins (also referred to as host defense peptides) are members of innate immune effectors, which intestinal epithelial cells secrete into the luminal environment, where they can kill enteropathogens. In addition to their bactericidal effects, antimicrobial proteins play other roles in the host, participating in innate immune mechanisms. For example, β-defensins can regulate the membrane barrier, including the maintenance of the mucus layer, chemokine production, and the proliferation and migration of epithelial cells [1–5]. However, the roles of antimicrobial proteins beyond direct killing are poorly defined in the context of enteropathogens.

The Regenerating islet-derived protein III β (RegIIIβ) is a member of the C-type lectin family [6], which harbors bactericidal activity toward certain Gram-negative and Gram-positive bacteria by recognizing the carbohydrate moieties of lipopolysaccharide and peptidoglycan [7–10]. RegIIIβ proteins are secreted from Paneth cells and intestinal epithelial cells into the gut lumen [10,11], where the production of RegIIIβ is dramatically increased during commensal bacterial colonization, pathogenic infection, and intestinal inflammation [7,10–12]. The expression of RegIII family proteins, including RegIIIβ and RegIIIγ, can be regulated by the activation of pattern recognition receptors, MyD88 signaling, specialized intracellular nucleotide-binding oligomerization domain-like (NOD-like) receptors, and IL-22 via STAT3 [13–17]. Notably, gastrointestinal infection with enteropathogenic bacteria can induce the expression of RegIII via IL-22 produced by Th17 cells [17–19]. Thus, RegIII family proteins, including RegIIIβ, are considered innate immune effectors during infections with enteropathogens that can induce inflammatory responses [7,12,20–22].

Infectious diarrhea is a global concern for human health [23,24] and is a major cause of morbidity and mortality, especially in developing countries [25]. *Salmonella enterica* serovar Typhimurium (*S*Tm) is a leading cause of infectious diarrhea [26,27]. Oral consumption of contaminated food and water allows *S*Tm to reach the intestinal tract. In the gut lumen, flagella-driven motility directs *S*Tm towards invasion sites, cooperatively inducing *S*Tm invasion [28]. The initial growth of *S*Tm also depends on flagellar motility, which efficiently induces gut inflammation [29]. Subsequent invasion of gut tissue by *S*Tm is accompanied by elevated inflammatory responses and diarrhea [30–32]. Gut inflammation provides a competitive advantage by outcompeting the resident gut microbiota and establishing a niche in the gut lumen [33–36]. Although accumulating evidence suggests the important roles of gut inflammation in gastrointestinal infection, the mechanism by which *S*Tm colonizes the gut lumen in the early stage of infection, when inflammation is mild, is predicted to differ from that in the later stage, which is accompanied by more severe gut inflammation [33,37]. Thus, it is far less understood how gut inflammation contributes to *S*Tm infectivity in the early stages of gastrointestinal infection.

Earlier work demonstrated the protective role of RegIIIβ, showing that this lectin inhibits intestinal translocation of *S*Tm in a mouse infection model with oral administration [20]. The protective mechanism of RegIIIβ does not rely on its bactericidal activity, but the molecular mode of action remains unclear. On the other hand, by using the streptomycin mouse model for *Salmonella* diarrhea [38,39], we have previously shown that RegIIIβ contributes to the persistent colonization of *S*Tm by repressing the regrowth of *Bacteroides* spp. in the intestinal tract, accompanied by alterations in the metabolic profile [40]. We here show that in the initial stage of infection of the streptomycin mouse model, RegIIIβ binds to *S*Tm independently of its bactericidal activity and facilitates gut colonization of *S*Tm by activating flagella-driven motility through maintenance of the membrane potential. This also resulted in enhanced invasion into epithelial cells by *S*Tm. Our findings provide novel insight into the roles of RegIIIβ in the early stages of gastrointestinal infection by *S*Tm.

## Results

### RegIIIβ binds to *S*Tm in the gut lumen, independently of its bactericidal effect

**Fig 1:**
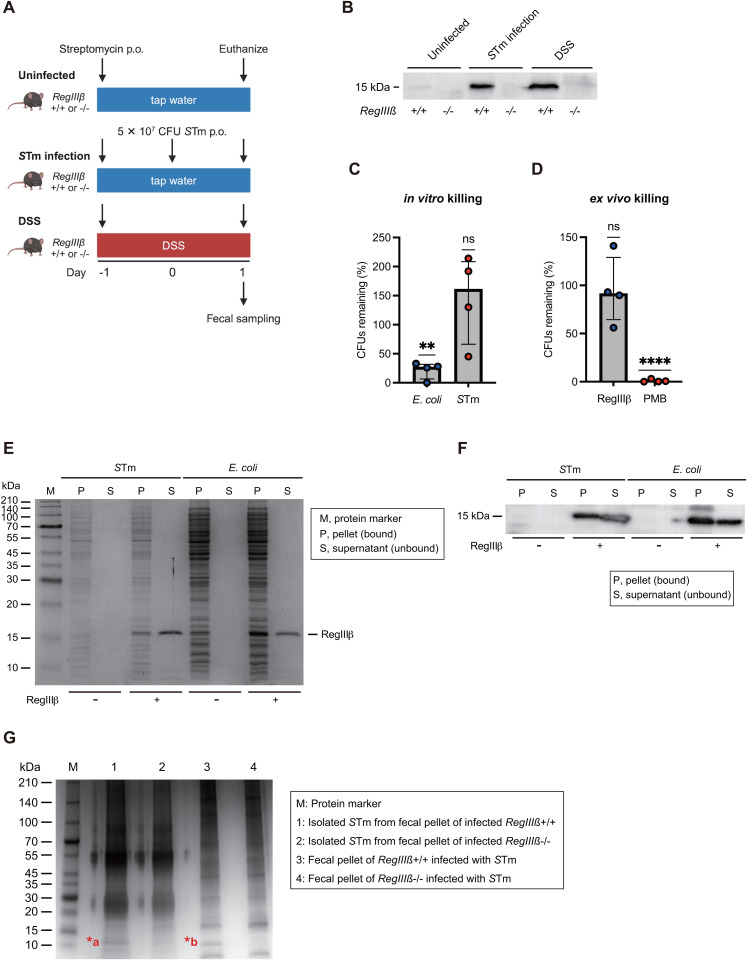
Induced RegIIIβ binds to *S*Tm in the gut, independently of its bactericidal activity. (A) Mouse experiment scheme. *RegIIIβ*^-/-^ mice or littermate controls were given either tap water or 2% DSS, followed by streptomycin treatment. Similarly, *RegIIIβ*^-/-^ mice or littermate controls given tap water were pretreated with streptomycin and infected with *S*Tm via oral gavage 24 h later. Mice were euthanized on day 2 post-streptomycin treatment or day 1 post-infection, and fecal samples were collected. (B) Protein levels of RegIIIβ in the fecal samples. RegIIIβ in the feces was analyzed by SDS-PAGE and Western blotting using anti-RegIIIβ antibodies. (C) *In vitro* killing by recombinant RegIIIβ. Mouse-isolated *E*. *coli* or *S*Tm were grown in LB broth and exposed to RegIIIβ (10 µM) for 30 min at 37°C. Bactericidal survival was quantified by dilution-plating on LB agar. Percentage of CFUs remaining after exposure to recombinant RegIIIβ. *n* is indicated by the number of dots. Data were obtained from four mice from four independent experiments. Bars, median with interquartile range. One-sample t test. *P* > 0.05 not significant (ns), *P* < 0.05 (^*^), *P* < 0.01 (^**^), *P* < 0.001 (^***^), *P* < 0.0001 (^****^). (D) *Ex vivo* killing by recombinant RegIIIβ. The microbiota was isolated from the feces of *RegIIIβ*^-/-^ mice, and directly exposed RegIIIβ (10 µM) or polymyxin B (PMB) (1 µg/ml) for 30 min at 37°C. Percentage of CFUs remaining after exposure to recombinant RegIIIβ or PMB. The killing effect shown here is on the *S*Tm within the microbiota. *n* is indicated by the number of dots. Data were obtained from 4 mice from two independent experiments. Bars, median with interquartile range. One-sample t test. *P* > 0.05 not significant (ns), *P* < 0.05 (^*^), *P* < 0.01 (^**^), *P* < 0.001 (^***^), *P* < 0.0001 (^****^). (E and F) *In vitro* binding of recombinant RegIIIβ. *S*Tm or *E*. *coli* grown to the logarithmic growth phase were incubated with RegIIIβ at 37°C for 15 min. The samples were centrifuged to separate the RegIIIβ-bound bacteria (*P*, pellet) or unbound RegIIIβ (*S*, supernatant), and analyzed by SDS-PAGE and Coomassie Brilliant Blue staining (E) or Western blotting using anti-RegIIIβ antibodies (F). (G) Gel electrophoresis of fecal samples and the isolated *S*Tm by anti-*Salmonella* O4 LPS antibody-conjugated beads from *RegIIIβ*^-/-^ mice or littermate controls. Bands marked by the asterisk (*a and *b) were eluted and subjected to mass spectrometry. The bands were identified as RegIIIβ.

The production of RegIIIβ was increased during the early stages of *S*Tm gastrointestinal infection and intestinal inflammation, as evidenced by our results showing that *S*Tm infection via oral gavage and dextran sodium sulfate (DSS) treatment in the streptomycin mouse model allowed for the detection of RegIIIβ protein in the fecal samples (Fig 1A and 1B). The results show that RegIIIβ is induced after oral gavage with *S*Tm infection or with DSS in the streptomycin model. In contrast, increased luminal levels of RegIIIβ were not observed in oral infection with *S*Tm in a mouse model without streptomycin pretreatment (S1A Fig). Likewise, earlier work with immunohistochemistry of intestinal tissue sections showed very weak RegIIIβ levels in non-inflamed intestinal tissue of avirulent *S*Tm-infected mice, whereas abundant RegIIIβ expression in the tissue was observed in mice with *S*Tm-induced gut inflammation [7]. Furthermore, fecal levels of *S*Tm were low at the early stage of gastrointestinal infection in this model, as evidenced by our results showing that *S*Tm loads were below detection limits in most mice (S1B Fig). Similar poor *S*Tm colonization was observed in the mesenteric lymph node, spleen, and liver (S1C–S1E Fig). Thus, this study investigates the role of RegIIIβ in *S*Tm infection using the streptomycin mouse model, as previous models without streptomycin pretreatment showed limited RegIIIβ expression and *S*Tm colonization.

We thus asked whether the produced RegIIIβ could kill *S*Tm in the gut. Recombinant RegIIIβ protein killed mouse-isolated *E*. *coli* (mEC-1), whereas the *S*Tm wild-type strain (WT) was tolerant to RegIIIβ-mediated killing (Fig 1C). Likewise, RegIIIβ did not kill *S*Tm from the feces of infected *RegIIIβ*^-/-^ mice, whereas the polymyxin B (PMB), a potent antimicrobial toward Gram-negative bacteria, displayed bactericidal effects on *S*Tm (Fig 1D). In line with previous studies [7,40], we thus found that *S*Tm in the gut can resist the RegIIIβ-mediated bactericidal effects. This is surprising, since earlier work showed that fast-growing *S*Tm cells are susceptible to RegIIIβ [9], and *S*Tm in the gut lumen is expected to grow rapidly [33,38,41]. Notably, however, *S*Tm appears to become resistant to RegIIIβ in the gut environment, for reasons that remain to be determined. These results also raise the possibility that RegIIIβ cannot bind to *S*Tm in the gut lumen since binding to target bacteria is a prerequisite for bacterial killing [8,9]. In contrast, we have demonstrated the binding ability of RegIIIβ to *S*Tm by examining the *in vitro* interaction between recombinant RegIIIβ protein and bacterial cells [8,9]. Consistent with this observation, RegIIIβ bound to both *S*Tm and *E*. *coli* (Fig 1E and 1F). It is notable that the results appear to contradict the hypothesis that RegIIIβ may not bind to *S*Tm. Thus, we next investigated whether RegIIIβ in the gut lumen could bind to *S*Tm. To this end, infected *S*Tm cells from the feces of *RegIIIβ*^-/-^ or littermate controls (*RegIIIβ*^+/+^) were isolated using anti-O4 *Salmonella* LPS antisera-immobilized beads and analyzed for *S*Tm-bound proteins. The isolate was separated by electrophoresis and analyzed with silver staining. An approximately 13-kDa band, which is proteolytically processed by trypsin or a trypsin-like protease *in vivo* [42], was found in samples from *RegIIIβ*^+/+^ mice but not in samples from *RegIIIβ*^-/-^ mice (Fig 1G). We thus subjected the bands to nano-liquid chromatography‒tandem mass spectrometry (nano-LC-MS/MS) and identified RegIIIβ (S1 Table). These results suggest that *S*Tm infection‒induced RegIIIβ binds to this bacterium in the gut lumen, independently of its killing effect.

### Binding of RegIIIβ to *S*Tm increases locomotion speed, resulting in enhanced invasiveness into epithelial cells

**Fig 2:**
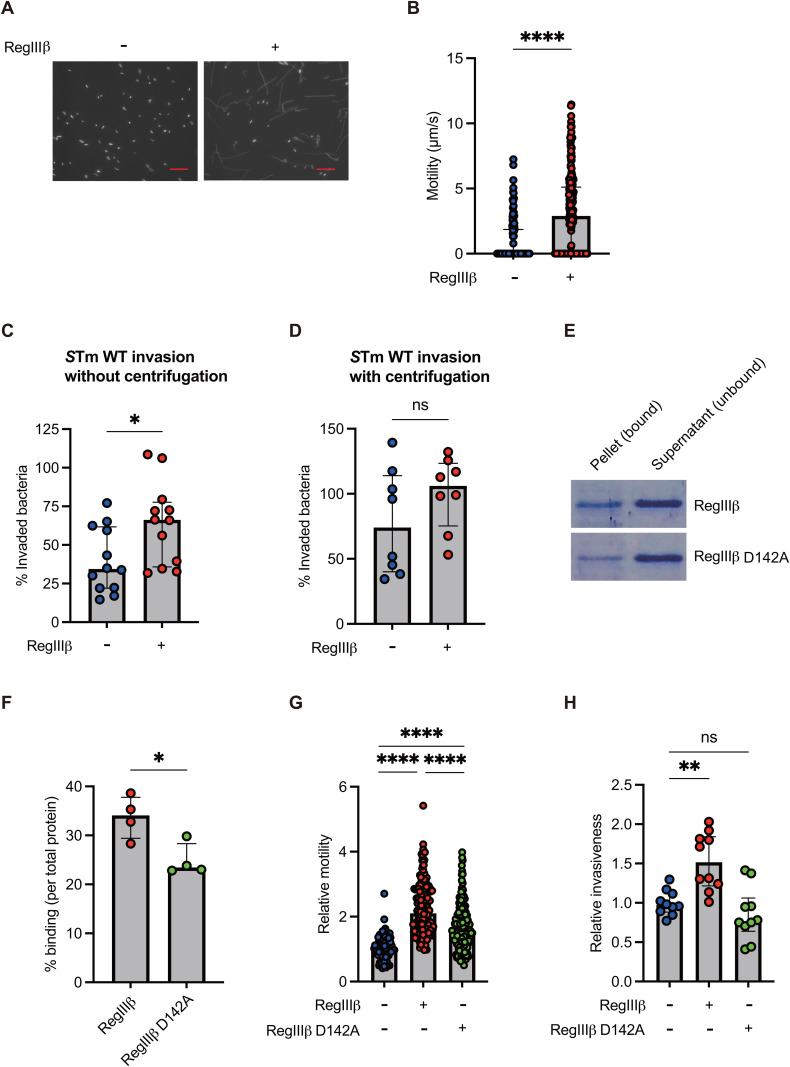
RegIIIβ increases locomotion speed of *S*Tm in the gut, leading to enhanced invasion into epithelial cells. (A) Microscopy images of *S*Tm expressing green fluorescent proteins. The *S*Tm strains were preincubated with recombinant RegIIIβ, placed on a glass slide, sealed under a glass coverslip, and imaged by fluorescence microscopy (exposure time: 2.6 s). Red scale bar, 10 µm. (B) Microscopy quantification of *S*Tm locomotor velocity of the experiment in panel A. *n* = 124 [RegIIIβ-], and 211 [RegIIIβ+]. Data were obtained from at least three independent experiments. Bars, median with interquartile range. Two-tailed Mann‒Whitney U test. *P* > 0.05 not significant (ns), *P* < 0.05 (^*^), *P* < 0.01 (^**^), *P* < 0.001 (^***^), *P* < 0.0001 (^****^). (C and D) Invasiveness into HeLa cells. *S*Tm was preincubated with recombinant RegIIIβ (10 µM) and added the monolayer cultures of HeLa cells. If needed, the centrifugation step was applied for close contact between *S*Tm cells and HeLa cells. Quantified invasiveness was determined by defining the input *S*Tm cells (inoculum) as 100%. *n* is indicated by the number of dots. Data were obtained from at least three independent experiments. Bars, median with interquartile range. Two-tailed Mann‒Whitney U test. *P* > 0.05 not significant (ns), *P* < 0.05 (^*^), *P* < 0.01 (^**^), *P* < 0.001 (^***^), *P* < 0.0001 (^****^). (E) Binding of RegIIIβ D142A to *S*Tm. *S*Tm grown up to the logarithmic growth phase were incubated with recombinant RegIIIβ D142A (10 µM) at 37°C for 15 min. The samples were centrifuged to separate the RegIIIβ-bound bacteria (pellet) or unbound RegIIIβ (supernatant), and analyzed by SDS-PAGE and Coomassie Brilliant Blue staining. (F) Quantitative analyses of the experiment in panel E. Quantified protein levels (%) of the pellet fractions (binding) were determined by defining the relative intensity of total fraction (pellet and supernatant) as 100%. *n* is indicated by the number of dots. Data were obtained from four independent experiments. Bars, median with interquartile range. Unpaired t test. *P* > 0.05 not significant (ns), *P* < 0.05 (^*^), *P* < 0.01 (^**^), *P* < 0.001 (^***^), *P* < 0.0001 (^****^). (G) Motility of *S*Tm preincubated with RegIIIβ or a D142A point-mutated RegIIIβ. Data were determined by defining the relative motility of *S*Tm with no addition with RegIIIβ as 1. *n* = 98 [RegIIIβ- RegIIIβ D142A-], 164 [RegIIIβ + RegIIIβ D142A-], and 165 [RegIIIβ- RegIIIβ D142A+]. Data were obtained from at least three independent experiments. Bars, median with interquartile range. A one-way ANOVA followed by Dunnett’s multiple comparisons test. *P* > 0.05 not significant (ns), *P* < 0.05 (^*^), *P* < 0.01 (^**^), *P* < 0.001 (^***^), *P* < 0.0001 (^****^). (H) Invasiveness into HeLa cells of *S*Tm. *S*Tm was preincubated with recombinant RegIIIβ or a D142A point-mutated RegIIIβ and added the monolayer cultures of HeLa cells. Data were determined by defining the relative invasion by *S*Tm with no addition with RegIIIβ as 1. *n* is indicated by the number of dots. Data were obtained from at least three independent experiments. Bars, median with interquartile range. A one-way ANOVA followed by Dunnett’s multiple comparisons test. *P* > 0.05 not significant (ns), *P* < 0.05 (^*^), *P* < 0.01 (^**^), *P* < 0.001 (^***^), *P* < 0.0001 (^****^).

To explore the role of RegIIIβ binding to *S*Tm, we microscopically observed *S*Tm cells in the presence of recombinant RegIIIβ proteins. GFP-expressing *S*Tm cells grown to the late-logarithmic phase were pelleted by centrifugation, washed with binding buffer (25 mM MES [pH 6.0], 25 mM NaCl), suspended in binding buffer, and incubated with RegIIIβ at a concentration of 10 µM. The binding buffer allows RegIIIβ to bind to *S*Tm efficiently [8,9]. Microscopic analysis using a long exposure time to visualize movement as tracks of fluorescent bacteria revealed that incubation with RegIIIβ activates flagella-driven locomotion of *S*Tm relative to the binding buffer without RegIIIβ (Fig 2A). Notably, *S*Tm in binding buffer appeared to be nonmotile or to locomote at low speed, likely due to the absence of an energy source. The addition of RegIIIβ to the binding buffer significantly increased the movement velocity of *S*Tm cells (Fig 2B). Based on these results, it was tempting to speculate that the accelerated movement from the addition of RegIIIβ increases the invasion of *S*Tm to host cells. Thus, we investigated the interaction of RegIIIβ-preincubated *S*Tm with epithelial cells. Preincubation with RegIIIβ led to enhanced invasiveness of *S*Tm (Fig 2C). In the experiments, a 3-hour incubation period may have multiple rounds of infection including bacterial invasion, bacterial reinfection, intracellular growth in invaded cells, and extracellular bacterial growth in cultured cell media. Thus, to more accurately assess the ability of RegIIIβ-dependent invasion, we also employed a shorter incubation period (1 hour) and confirmed the enhanced invasiveness by RegIIIβ preincubation (S2 Fig). Furthermore, the RegIIIβ-enhanced invasion was likewise observed in the human intestinal epithelial cell line, Caco-2 (S3 Fig).

Next, to explore the possible link between enhanced motility and increased invasion, we examined the effect of RegIIIβ binding in a nonmotile *S*Tm invasion assay. RegIIIβ did not enhance the invasiveness of the nonmotile *S*Tm (∆*fliGHI*) (S4 Fig). Next, we used centrifugation in the *S*Tm invasion assay to facilitate *S*Tm and HeLa cell contact, bypassing flagellar motility. This method showed that RegIIIβ binding does not enhance invasiveness when direct cell contact is established even in the absence of motility (Fig 2D). Since the invasion process involves both flagellar motility and T3SS-dependent entry [43], the results indicate that RegIIIβ does not significantly affect T3SS. On the other hand, several pathways for the T3SS-independent cell entry, such as Rck [44], PagN [45], and SiiE [46] have been identified. It is notable that RegIIIβ-enhanced motility leads to an increase in the activity of T3SS-independent invasion, as evidenced by the results that RegIIIβ positively influenced the invasion capacity of a T3SS-deficient *S*Tm (S5 Fig). Also, the positive effect by RegIIIβ was canceled by centrifugation. Overall, RegIIIβ boosts flagellar motility, resulting in increased invasiveness involved in both T3SS-dependent and -independent mechanisms.

Finally, we examined the impact of RegIIIβ binding on intracellular replication after invasion. We found that RegIIIβ binding had a positive effect on subsequent intracellular replication, as evidenced by the faster replication of RegIIIβ-pretreated *S*Tm cells compared to untreated controls (S6 Fig). Thus, we concluded that the enhanced locomotion speed contributes to the invasiveness of *S*Tm into host cells. These results raise the possibility that RegIIIβ-enhanced invasiveness influences bacterial replication within epithelial cells.

To more clearly define the causal link between the binding of RegIIIβ to *S*Tm and accelerated movement and invasion, we used a D142A point-mutated RegIIIβ protein that has reduced binding capacity toward substrate lipid A [9]. The D142A variant of RegIIIβ bound with reduced efficiency compared to the WT protein (Fig 2E). Approximately 32.4% of the binding capacity was reduced in RegIIIβ D142A compared to RegIIIβ WT (Fig 2F). Thus, we measured locomotion speed upon the addition of RegIIIβ D142A. This addition slightly increased the locomotion velocity of *S*Tm, whereas the effect of RegIIIβ D142A was significantly lower than that of RegIIIβ WT (Fig 2G). Similarly, RegIIIβ D142A had no effect on the enhanced invasion into HeLa cells (Fig 2H). These results indicate that the binding of RegIIIβ to *S*Tm enhances motility and invasiveness.

We next asked whether RegIIIβ affects the flagellar motility of enteric bacterial pathogens. We specifically focused on *E*. *coli* strain LF82 [47] as this pathobiont is a flagellated motile strain and has the ability to invade epithelial cells and colonize the inflamed gut as well as *S*Tm [48–50]. Unlike *S*Tm, preincubation with RegIIIβ had no effect on flagellar motility of LF82 (S7A Fig). Rather, LF82 cells aggregated upon the addition of RegIIIβ. Thus, we hypothesized that LF82 has not evolved resistance to RegIIIβ. As expected, RegIIIβ displayed bactericidal activity against LF82 in a concentration-dependent manner (S7B Fig). Furthermore, we confirmed growth kinetics in the presence of RegIIIβ. *S*Tm and *E*. *coli* strain LF82 were mixed and incubated with 10 µM RegIIIβ. Killing effects of RegIIIβ on individual strains were monitored by determining bacterial loads via selective plating. Bacterial loads of LF82 significantly decreased when incubated along with RegIIIβ, whereas growth of *S*Tm WT strain SL1344 remained unaffected by the presence of RegIIIβ (S7C Fig). These findings indicate that RegIIIβ binding-dependent enhanced motility may be specific to *S*Tm cells. Moreover, this may support the hypothesis that *S*Tm has become tolerant to RegIIIβ through evolution while simultaneously acquiring the capacity to exploit RegIIIβ to increase locomotion speed.

### RegIIIβ activates the membrane potential and increases ATP production

**Fig 3:**
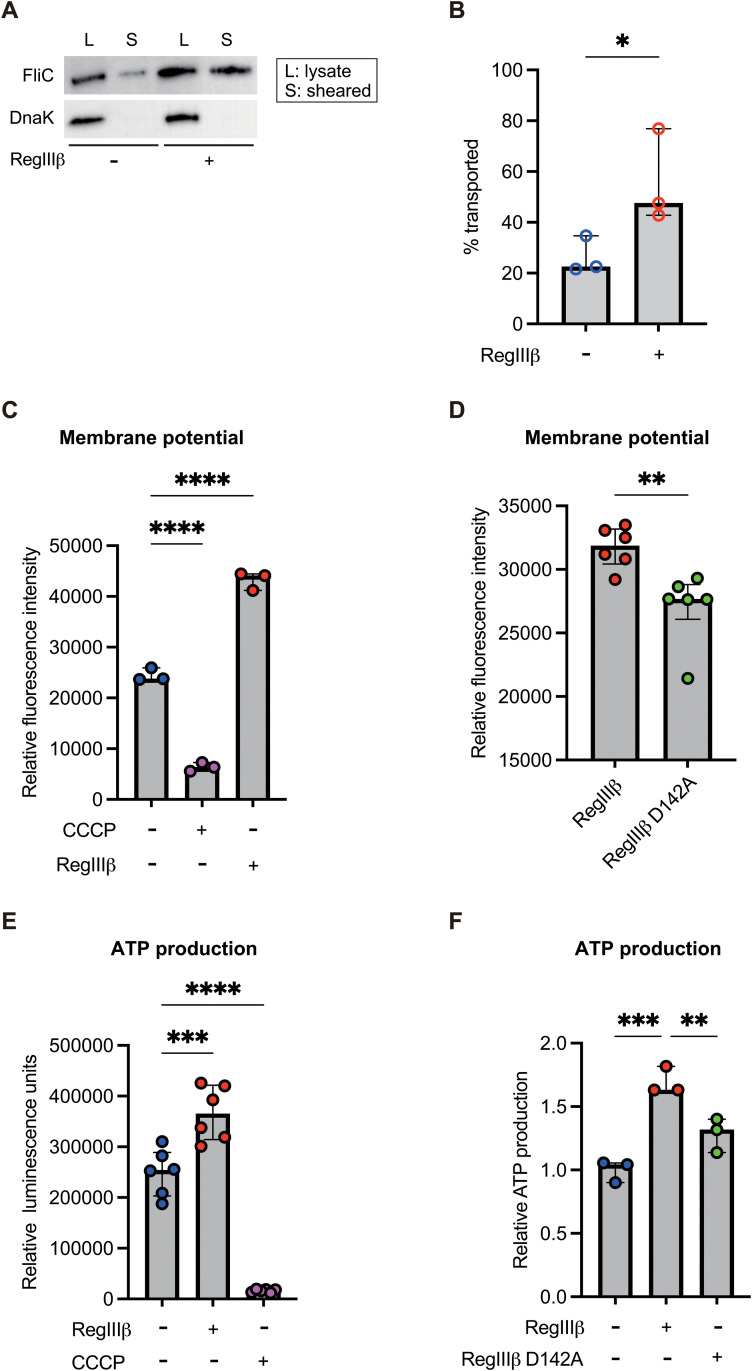
RegIIIβ binding maintains the membrane potential, leading to increased ATP production. (A) Intracellular and transported FliC of the RegIIIβ-preincubated *S*Tm. Samples of lysate (*L*, intracellular expression) and sheared (*S*, transported) were subjected to SDS-PAGE and analyzed by Western blotting with anti-FliC and anti-DnaK antibody. (B) Quantification of transported FliC levels. The band intensity was analyzed using ImageJ software. *n* is indicated by the number of dots. Data were obtained from three independent experiments. Bars, median with interquartile range. Paired t test. *P* > 0.05 not significant (ns), *P* < 0.05 (^*^), *P* < 0.01 (^**^), *P* < 0.001 (^***^), *P* < 0.0001 (^****^). (C and D) Fluorescence intensities of DiSC_3_(5) in the RegIIIβ-bound *S*Tm. Logarithmically grown cultures of *S*Tm were resuspended in binding buffer (25 mM MES [pH 6.0], 25 mM NaCl), followed by incubation with 1 µM DiSC_3_(5). After the incubation, the samples were added with 1 mM CCCP or 10 µM recombinant RegIIIβ or a D142A point-mutated RegIIIβ. DiSC_3_(5) fluorescence intensities were quantified using a fluorescence spectrometer. *n* is indicated by the number of dots. Data were obtained from at least three independent experiments. Bars, median with interquartile range. Two-tailed Mann‒Whitney U test. *P* > 0.05 not significant (ns), *P* < 0.05 (^*^), *P* < 0.01 (^**^), *P* < 0.001 (^***^), *P* < 0.0001 (^****^). (E and F) Intracellular ATP levels. The levels of intracellular ATP were measured by a luciferin-luciferase bioluminescence assay. *S*Tm grown to the logarithmic growth phase were resuspended in binding buffer (25 mM MES [pH 6.0], 25 mM NaCl), followed by incubation with 1 mM CCCP or 10 µM recombinant RegIIIβ or a D142A point-mutated RegIIIβ. Luminescence units (E) or relative values to no addition control (F) were determined. *n* is indicated by the number of dots. Data were obtained from at least three independent experiments. Bars, median with interquartile range. A one-way ANOVA followed by Dunnett’s multiple comparisons test. *P* > 0.05 not significant (ns), *P* < 0.05 (^*^), *P* < 0.01 (^**^), *P* < 0.001 (^***^), *P* < 0.0001 (^****^).

How does RegIIIβ binding lead to increased flagella-driven locomotion speed? We first investigated the expression levels of flagellar genes by reverse transcription (RT)-quantitative PCR (qPCR). Transcript levels of selected class 1 flagellar genes (*flhD* and *flhC*) and class 2 flagellar genes (*fliA* and *flgB*) were similar between the RegIIIβ-preincubated *S*Tm and the untreated control (S8A and S8B Fig). In contrast, transcript levels of *fliC* and *fljB*, class 3 flagellar genes, in the RegIIIβ-preincubated *S*Tm were decreased compared to those in the untreated control (S8C Fig). Thus, we next investigated the intracellular (lysate) and surface-transported levels (sheared) of FliC proteins. Notably, transported FliC levels were higher in RegIIIβ-preincubated *S*Tm than in the untreated control (Fig 3A and 3B). Intracellular levels of DnaK, an internal control, were equivalent between the two conditions (Fig 3A). Furthermore, transported DnaK was not detected in either sample, indicating that the sheared FliC proteins were not contaminated by intracellular proteins. These results indicate that the enhanced locomotion speed of RegIIIβ-preincubated *S*Tm is not due to increased transcription of flagellar genes. On the other hand, the results raise the possibility that the transport capacity for substrates such as FliC flagellin may be enhanced in RegIIIβ-preincubated *S*Tm.

Secretion of substrates via the flagellum mainly depends on the proton motive force (PMF) as a source of energy [51]. PMF also supplies fuel into the flagellum, allowing the flagellar motor to rotate and enabling swimming motility [52]. Therefore, we hypothesized that the binding of RegIIIβ to *S*Tm may lead to an increase in PMF by affecting membrane integrity, thereby enhancing flagella-driven motility. To this end, we investigated whether RegIIIβ binding influences the membrane potential using the voltage-sensitive dye DiSC_3_(5), which can be used to monitor the membrane potential [53], as confirmed by results showing that carbonyl cyanide m-chlorophenylhydrazone (CCCP), an ionophore, reduced DiSC_3_(5) signal intensity (Fig 3C). Incubation with RegIIIβ led to increase in membrane potential (Fig 3C). Thus, these results highlight the crucial role of RegIIIβ in increasing membrane potential and enhancing flagellar motility, which significantly increases *S*Tm’s invasiveness into host cells. In contrast, the membrane potential of *S*Tm preincubated with RegIIIβ D142A was reduced compared to that of RegIIIβ-bound *S*Tm (Fig 3D).

Based on previous reports on the interaction between RegIIIβ and *S*Tm cells [8], we hypothesized that RegIIIβ binding facilitates outer membrane permeability, thereby enhancing ion entry and causing a transient increase in membrane potential. Our experiments confirmed this hypothesis, demonstrating that incubation with RegIIIβ in LB medium enhances flagellar motility by promoting ion entry (S9A and S9B Fig), which in turn increases the invasiveness of *S*Tm into epithelial cells (S9C Fig).

We next asked whether the maintenance of membrane potential by RegIIIβ binding affects ATP production, since membrane potential acts as a key energy reserve for ATP synthesis. As expected, RegIIIβ binding promoting ion entry led to an increase in ATP production, whereas CCCP-mediated dissipation of membrane potential dramatically reduced ATP levels (Fig 3E). In contrast, ATP production in RegIIIβ D142A-bound *S*Tm was reduced compared to that in the RegIIIβ-bound control (Fig 3F). Altogether, these results indicate a possible link between RegIIIβ binding‒dependent flagellar movement and membrane potential.

### Intestinal luminal RegIIIβ facilitates *S*Tm colonization and enteropathy in the initial stage of infection by activating flagella-driven motility

**Fig 4:**
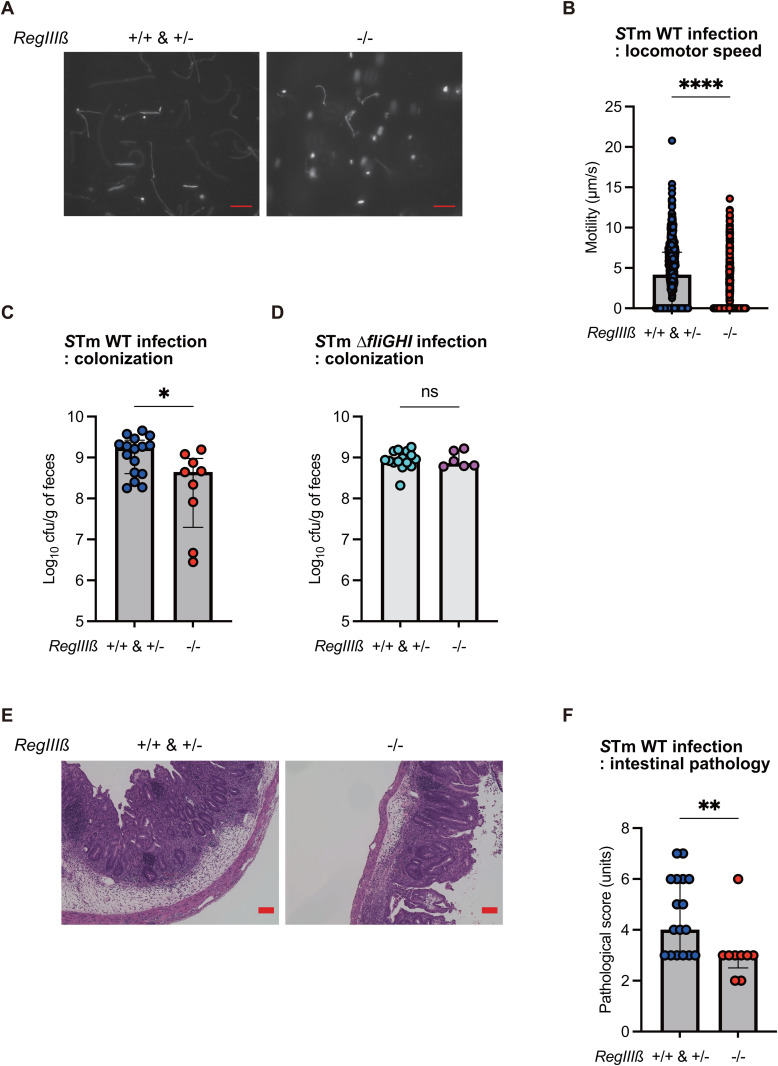
RegIIIβ increases infectivity of the early stages of gastrointestinal infection of *S*Tm by activating flagella-driven motility. (A) *S*Tm movement in the feces. Streptomycin-treated *RegIIIβ*^-/-^ mice or littermate controls (*RegIIIβ*^+/+^ and *RegIIIβ*^+/-^) were infected by oral gavage for 1 day with GFP-expressing *S*Tm. Fecal pellets were collected, and then resuspended with PBS. The resulting *S*Tm cells were immediately observed by fluorescence microscopy (exposure time: 2.6 s). Red scale bar, 10 µm. (B) Microscopy quantification of *S*Tm locomotor velocity of the experiment in panel A. *n* = 783 [*RegIIIβ*^*+/+*^ & *RegIIIβ*^*+/-*^], and 916 [*RegIIIβ*^*-/-*^]. Data were obtained from at least three independent experiments. Bars, median with interquartile range. Two-tailed Mann‒Whitney U test. *P* > 0.05 not significant (ns), *P* < 0.05 (^*^), *P* < 0.01 (^**^), *P* < 0.001 (^***^), *P* < 0.0001 (^****^). (C-F) Streptomycin-treated *RegIIIβ*^-/-^ mice or littermate controls (*RegIIIβ*^+/+^ and *RegIIIβ*^+/-^) were infected by oral gavage for 1 day with *S*Tm wild-type strain or ∆*fliGHI* mutant. Bacterial loads of *S*Tm wild-type strain (C) (mice = 16 [*RegIIIβ*^+/+^ and *RegIIIβ*^+/-^] or 9 [*RegIIIβ*^*-/-*^]) or ∆*fliGHI* mutant (D) (mice = 15 [*RegIIIβ*^+/+^ and *RegIIIβ*^+/-^] or 6 [*RegIIIβ*^*-/-*^]) in the feces were determined by serial-dilution and plating on MacConkey agar plate. (E) Light microscopy images of cecum tissue (H&E staining) of *RegIIIβ*^-/-^ mice or littermate controls (*RegIIIβ*^+/+^ and *RegIIIβ*^+/-^) infected with *S*Tm WT. Red scale bar, 100 µm. (F) Cecal pathology was scored in H&E-stained cecal tissue section of *RegIIIβ*^-/-^ mice or littermate controls infected with *S*Tm wild-type strain. *n* is indicated by the number of dots. Data were obtained from at least two independent experiments. Bars, median with interquartile range. Two-tailed Mann‒Whitney U test. *P* > 0.05 not significant (ns), *P* < 0.05 (^*^), *P* < 0.01 (^**^), *P* < 0.001 (^***^), *P* < 0.0001 (^****^).

We next asked whether the effects of RegIIIβ binding on locomotion speed and cell invasion by *S*Tm contribute to gastrointestinal infection caused by this bacterium. Feces were collected from the streptomycin model *RegIIIβ*^-/-^ mice or wild-type littermate controls (*RegIIIβ*^+/+^ and *RegIIIβ*^+/-^) on day 1 post-infection with *S*Tm. These samples were homogenized in cold PBS and left on ice to allow food components to sediment. The resulting supernatant was then observed using fluorescence microscopy to assess the luminal locomotion speed derived from flagellar motility of *S*Tm. Microscopic analysis of the feces revealed that *S*Tm cells in the gut of the littermate controls moved faster than those from *RegIIIβ*^-/-^ mice, although the motility was low, possibly by prior treatment of feces (Fig 4A). The locomotion velocity of the *S*Tm from the littermate controls was greater than that from *RegIIIβ*^-/-^ mice (Fig 4B). Thus, we next investigated whether this enhanced motility contributes to the infectivity of *S*Tm. Colonization levels of *S*Tm on day 1 post-infection in the littermate controls were higher compared to those in *RegIIIβ*^-/-^ mice (Fig 4C). The increased colonization levels due to the presence of luminal RegIIIβ were not observed in the context of the aflagellated mutant lacking the *fliGHI* gene (Fig 4D). In addition, a histopathologic scoring [38] showed that *S*Tm-infected *RegIIIβ*^-/-^ mice exhibited lower grade mucosal inflammation compared to the littermate controls (Fig 4E and 4F). To further highlight the critical role of RegIIIβ in facilitating *S*Tm colonization, we next performed mixed infection experiments using the *S*Tm WT and ∆*fliGHI* strains. Streptomycin-pretreated *RegIIIβ*^-/-^ mice or wild-type littermate controls (*RegIIIβ*^+/+^) were infected with a 1:1 mixture of the WT and ∆*fliGHI* via oral gavage. On the first day post-infection, we quantified bacterial loads in the feces and calculated competitive indices (CI). The CI values (WT/∆*fliGHI*) in *RegIIIβ*^-/-^ mice were significantly lower than those in wild-type littermate controls (S10 Fig), underscoring the substantial contribution of RegIIIβ-enhanced flagellar motility to early-stage gut colonization. Collectively, these results position RegIIIβ as a host factor that fortifies the *S*Tm invasion of the gut in the early stages of gastrointestinal infection by activating flagella-driven motility.

## Discussion

It may be reasonable to consider that RegIIIβ acts as an innate immune effector, as evidenced by the fact that RegIIIβ is induced in response to pathogen infection and has bactericidal activities [54]. Unexpectedly, *S*Tm was resistant to the killing activity of RegIIIβ in the gut lumen; nevertheless, the antimicrobial could bind to *S*Tm. Since direct binding to the target is a prerequisite for killing by RegIIIβ [8,9], our findings showing the binding to *S*Tm appear contradictory. On the other hand, the findings prompt us to speculate that the binding of RegIIIβ may have previously unrecognized roles in gastrointestinal infection by *S*Tm. Thus, our findings reveal that RegIIIβ fortifies the infectivity of *S*Tm in the early stages of gastrointestinal infection. Binding of RegIIIβ enhances flagella-driven locomotion by maintaining membrane potential and increasing ATP production. This leads to enhanced invasiveness to epithelial cells and colonization in the gut lumen. Therefore, we conclude that *S*Tm has become able to exploit RegIIIβ to efficiently infect host cells as part of its adaptation to resist the antimicrobial. However, we are far from fully understanding the effects of RegIIIβ binding, which will be a fascinating topic for future work. We expect that this study will open the door to future research revealing new roles of RegIII in enteric infection.

Our previous studies indicated that *S*Tm cells in the logarithmic growth phase are vulnerable to RegIIIβ *in vitro* [9]. However, *in vivo* studies show *S*Tm cells from murine feces resist RegIIIβ, suggesting a potential adaptive mechanism that enhances their flagellar motility and colonization ability. The effects of RegIIIβ binding from *in vivo* experiments (animal models) have been extremely helpful in identifying mechanisms that can affect the pathogen-host interaction. For instance, *in vivo* studies have shown that RegIIIβ can significantly promote *S*Tm load in the gut, which is not observed in *in vitro* settings [40]. In contrast to *in vitro* experiments, *in vivo* experiments offer the option to focus on the molecular pathways in the infection stages of interest, such as the immune response activation. It is notable that the identified mechanisms might be unimportant during *in vitro* experiments, as RegIIIβ conversely kills the fast-growing *S*Tm *in vitro*. Indeed, data from *in vitro* experiments will help design *in vivo* studies to understand pathogen-host interaction; however, limitations as seen in this study are inherent to any *in vivo* experiments. This research could pave the way for novel treatments targeting bacterial infections by exploiting the adaptive mechanisms of pathogens, potentially leading to more effective therapies.

How is *S*Tm adapted to the bactericidal effect of RegIIIβ in the evolutionary process? Our previous work showed that LPS counteracts the killing effects of RegIIIβ through steric hindrance that prevents RegIIIβ from accessing lipid A, its binding substrate [8]. In contrast, RegIIIβ normally can kill *E*. *coli* harboring similar LPS to *S*Tm [7]. Likewise, an enteric pathobiont, *E*. *coli* strain LF82 was susceptible to RegIIIβ. In addition, RegIIIβ did not enhance the flagellar motility of LF82, despite its flagellated nature, indicating that this enhancement is specific to *S*Tm. Given our findings that incubation with RegIIIβ induces agglutination of LF82, it is reasonable to hypothesize that tolerance to the bactericidal effect of RegIIIβ may be linked to the enhanced flagellar motility. Notably, our findings also indicated that RegIIIβ binds to *S*Tm in murine feces, independently of its bactericidal effect. These facts suggest that steric hindrance may not account for all of the *S*Tm tolerance to RegIIIβ. In addition to LPS, the robustness of the outer membrane also contributes to the resistance of *S*Tm to RegIIIβ, as evidenced by the results that the detergent Triton X-100 sensitizes *S*Tm to RegIIIβ [8]. Thus, it is reasonable to hypothesize that in susceptible bacteria such as *E*. *coli*, RegIIIβ can traverse the outer membrane and gain access to the inner membrane, a probable target for killing, whereas *S*Tm can withstand the insult of RegIIIβ by possessing a robust outer membrane. Conversely, *S*Tm exploits RegIIIβ for its virulence trait. Our data suggest that the RegIIIβ-bound form of *S*Tm helps maintain the membrane potential, which is critical for energizing flagellar rotation and motility. We do not yet know the molecular mechanism by which RegIIIβ influences the membrane potential. In contrast, binding of RegIIIβ to lipid A may be a prerequisite for the enhanced membrane potential, as evidenced by our findings that RegIIIβ D142A with reduced binding activity was less effective in increasing membrane potential. Collectively, one may imagine that the RegIIIβ-induced membrane potential leads to alterations in PMF, which is composed of the sum of membrane potential and the transmembrane proton gradient (∆pH).

The bacterial flagellum is one of the critical systems responsible for bacterial motility [55]. The motility is definitely driven by an artificial PMF [56]. The flagellar T3SS (fT3SS) of *S*Tm is equipped with a dual-fuel protein export engine that makes use of proton (H^+^) and sodium ion (Na^+^) as the coupling ions, which results in a membrane voltage sensor to drive flagellar protein export and power flagellar motor rotation [57–59]. Therefore, it is reasonable to hypothesize that RegIIIβ increases the availability of the coupling ions such as H^+^ and Na^+^ by enhancing ion entry across the outer membrane, as evidenced by our findings that RegIIIβ binding leads to an increase in the membrane potential. Remarkably, impaired sodium absorption is one of the pathologies in patients with inflammatory bowel disease [60,61]. Furthermore, changes in the ionic milieu, especially sodium ions, in the bowel play a pivotal role in inducing gut inflammation and colitis [62,63]. Since *S*Tm employs the Na^+^-coupled protein exporter under the specific condition [58,64], the facts allow us to imagine that the increased availability of Na^+^ in the inflamed gut due to *S*Tm infection may facilitate RegIIIβ’s effect on increased flagellar motility. Of note, RegIIIβ binding to *S*Tm also increased ATP production, a process for which increased PMF is also essential. To our best knowledge, this study is the first to reveal a host protein enhancing the locomotion speed of a bacterium. Since flagellar motility and chemotaxis are common traits among bacterial pathogens, including *S*Tm and are critical for successful infection and host colonization [65,66], our findings bring new insight into *Salmonella* pathogenesis exploiting intestinal inflammation.

## Materials and methods

### Ethics statement

Animal protocols were reviewed and approved by the Kitasato University Institutional Animal Care and Use Committee (Permit Number: 23–24 and 24–8).

### Mice

C57BL/6 (wild-type, *RegIIIβ*^+/+^) mice, *RegIIIβ*^+/-^ mice and *RegIIIβ*^-/-^ mice were bred in a specific pathogen-free facility at Department of Pharmacy, Kitasato University. All experiments employed littermate control and the knockout mice.

### Bacterial strains, growth media and bacterial growth

All bacterial strains used in this study are listed in S2 Table. Bacteria were routinely grown in Luria-Bertani (LB) broth at 37°C with agitation (160 rpm) or LB agar, supplemented with 50 µg/ml streptomycin, or 10 µg/ml chloramphenicol when needed.

### Mouse infection experiments

Mouse infection models, referred to as “the streptomycin mouse model”, were performed as previously described [38,40]. Mice were treated with oral dose of 25 mg streptomycin 24 h prior to infection and infected with 5 × 10^7^ CFU *S*Tm by oral gavage. Mixed infection experiments were performed as previously described [67]. Briefly, an equal mixture of bacterial cultures (totaling 1 × 10^8^ CFU) was administered via oral gavage. The competitive indices (CI) were determined by calculating the ratio of *S*Tm WT strain populations to their corresponding mutant derivatives and normalizing the ratio to the initial inoculum. If needed, mice were treated with a 2% (wt/vol) DSS (dextran sulfate sodium, molecular mass, 5000 Da) in drinking water to induce gut inflammation. Mice were euthanized on day 1 post-infection by cervical dislocation. Collected fecal pellets or cecal content were homogenized in sterile phosphate-buffered saline (PBS), and for evaluation of *S*Tm colonization, serially diluted, plated on MacConkey agar supplemented with 50 µg/ml streptomycin. To verify gut inflammation levels, 4% formaldehyde-fixed parts of cecal tissue were embedded in paraffin, and sections were cut and stained with hematoxylin and eosin (H&E). Cecal pathology was evaluated using H&E-stained sections and the following histopathological scoring scheme [38]. **(i) Submucosal edema.** Scoring for submucosal edema was defined as follows: 0, no pathological changes; 1, mild edema (submucosal edema: < 0.20 mm wide and <50% of the diameter of the entire intestinal wall); 2, moderate edema (submucosal edema: 0.21 to 0.45 mm wide and 50–80% of the diameter of the entire intestinal wall); 3, profound edema (submucosal edema: > 0.46 mm wide and >80% of the diameter of the entire intestinal wall). **(ii) Polymorphonuclear granulocyte (PMN) infiltration into the lamina propria.** PMN in the lamina were enumerated in 10 high-power fields (magnification, × 400; field diameter, 420 µm), and the average number of PMN per high-power field was calculated. Scoring was defined as follows: 0, fewer than 5 PMN per high-power field; 1, 5–20 PMN per high-power field; 2, 21–60 PMN per high-power field; 3, 61–100 PMN per high-power field; 4, more than 100 PMN per high-power field. **(iii) Goblet cells.** The average number of goblet cells per high-power field (magnification, × 400) was determined from 10 different regions of the cecal epithelium. Scoring was defined as follows: 0, > 28 goblet cells per high-power field (magnification, × 400); 1, 11–28 goblet cells per high-power field; 2, 1–10 goblet cells per high-power field; 3, < 1 goblet cell per high-power field. **(iv) Epithelial integrity.** Epithelial integrity was scored as follows: 0, no pathological changes detectable in 10 high-power fields (magnification, × 400); 1, epithelial desquamation; 2, erosion of the epithelial surface (gaps of 1–10 epithelial cells per lesion); 3, epithelial ulceration (gaps of more than 10 epithelial cells per lesion); at this stage, granulation tissue was generally present below the epithelium.

The pathological score for each tissue sample was determined by adding the averaged scores described above, and the total score indicated the following levels of inflammation: 0, intact intestine without any signs of inflammation; 1–2, minimal signs of inflammation (this level of inflammation is generally not considered a sign of disease); 3–4, slight inflammation; 5–8, moderate inflammation; 9–13, profound inflammation.

### Antibody generation

A polyclonal rabbit anti-RegIIIβ antibody was produced by immunization with the RegIIIβ peptide (PSTALDRAFC).

### Analysis for intestinal expression of RegIIIβ

A 25 mg fecal pellet was resuspended in 500 µl of PBS, mixed with SDS-PAGE sample buffer and boiled, followed by centrifugation. The resulting supernatant was separated by SDS-PAGE followed by Western blotting using the polyclonal anti-RegIIIβ antibody. The RegIIIβ was detected by chemiluminescent measurement.

### Purification of recombinant RegIIIβ

Recombinant RegIIIβ was prepared as previously described [8,9]. *E*. *coli* strain BL21 (DE3) harboring pET11a-RegIIIβ or pET11a-RegIIIβ D142A was induced with 0.5 mM IPTG for 4 h, and cells were harvested by centrifugation. Cells were washed with PBS and resuspended in inclusion body wash buffer (20 mM Tris-HCl, 10 mM EDTA, 0.01% Triton X-100). Cells were sonicated, and inclusion bodies containing RegIIIβ were sedimented by centrifugation. Purified inclusion bodies were resuspended in denaturing buffer (7 M guanidine-HCl, 0.15 M reduced glutathione, 2 mM EDTA, 0.1 M Tris-HCl [pH 8.0]) and incubated for 2 h. After centrifugation to remove insoluble material, the supernatant was diluted slowly into ice-cold refolding buffer (0.5 M arginine-HCl, 0.6 mM oxidized glutathione, 50 mM Tris-HCl [pH 8.0]), followed by overnight incubation. Insoluble material was removed by centrifugation, and the supernatant was concentrated by ultrafiltration using a pressure-mixing ultrafiltration machine (ADVANTEC) and an Amicon Ultra centrifugal filter, 10 kDa MWCO (Millipore). The concentrate was dialyzed three times against binding buffer (25 mM MES [pH 6.0], 25 mM NaCl), and insoluble material was removed by centrifugation. High purity of the prepared RegIIIβ was confirmed by analyzing by SDS-PAGE and Coomassie Brilliant Blue (CBB) staining. Furthermore, the bactericidal activity against the susceptible *E*. *coli* was confirmed by *in vitro* killing assay.

### *In vitro* killing assay

Bacteria grown to the middle- or late-logarithmic growth phase in LB broth, which has been shown to confer RegIIIβ tolerance in the case of *S*Tm [9], were washed and resuspended in binding buffer (25 mM MES [pH 6.0], 25 mM NaCl). The resulting bacteria were exposed to 10 µM RegIIIβ at 37°C for 30 min, and then plated on selective LB media. CFUs remaining (%) was evaluated by comparing the effect in the buffer control as 100%.

### *Ex vivo* killing assay

*Ex vivo* killing assay was performed as previously described [40]. Shortly, fecal pellets were collected from *RegIIIβ*^-/-^ mice infected with *S*Tm, homogenized in cold PBS and left on ice to sediment food component. The resulting supernatant was centrifuged to concentrate the microbiota, and the pellet was resuspended in binding buffer (25 mM MES [pH 6.0], 25 mM NaCl) and exposed to 10 µM RegIIIβ at 37°C for 30 min. Bacteria were then plated on MacConkey agar plate supplemented with 50 µg/ml streptomycin, and CFUs remaining (%) was normalized for the control in which binding buffer was incubated instead of the RegIIIβ as 100%.

### *In vitro* bacterial binding assay

Bacterial binding assay was performed as previously described [9]. Briefly, bacteria grown up to the logarithmic growth phase in LB broth were washed with binding buffer (25 mM MES [pH 6.0], 25 mM NaCl), and incubated with 10 µM RegIIIβ for 15 min at 37°C. After the incubation, centrifugation was performed, and the supernatant was transferred to a fresh tube, mixed with SDS-PAGE sample buffer. In contrast, the pellet was washed once, and resuspended with binding buffer, mixed with SDS-PAGE sample buffer. The samples were boiled for 5 min, and the supernatant was subjected to SDS-PAGE and analyzed by CBB staining and Western blotting using anti-RegIIIβ antibodies.

### Isolation of *S*Tm from murine feces and nano-LC-MS/MS

Fecal pellets were collected from *RegIIIβ*^-/-^ mice or the littermate controls (*RegIIIβ*^+/+^) infected with *S*Tm, and homogenized in cold PBS and left on ice. The resulting supernatant was centrifuged, and the pellet was resuspended in binding buffer (25 mM MES [pH 6.0], 25 mM NaCl). The resuspended samples were incubated with anti-O4 *Salmonella* LPS antibody for 37°C for 20 min. After the incubation, binding buffer-equilibrated MagnaBind Goat Anti-Rabbit IgG Beads (Thermo Fischer Scientific) were added, incubated at 4°C for 20 min. The beads were washed with binding buffer three times, resuspended in binding buffer and mixed with SDS-PAGE sample buffer. The samples were boiled for 5 min, and the supernatant was resolved on a 4%-15% SDS-PAGE gel and stained using EzStain Silver (ATTO). For protein identification, protein bands were excised and incubated with trypsin. The recovered peptides were desalted in ZipTip C18 (Merck Millipore), and analyzed by nano-LC-MS/MS (DiNa HPLC [high-pressure liquid chromatography] system, Techno Alpha, Tokyo, Japan; QSTAR XL hybrid LC-MS/MS system, Thermo Fischer Scientific). Mass data acquisitions were piloted using Mascot software.

### Analysis of bacterial movement with microscopy

Bacterial movement was analyzed using microscopy as previously described [68]. Shortly, GFP-expressing *S*Tm or *E*. *coli* resuspended in binding buffer (25 mM MES [pH 6.0], 25 mM NaCl) was incubated with 10 µM RegIIIβ for 15 min at 37°C. The samples were placed on a glass slide and sealed with a glass coverslip. The samples were observed with a Zeiss Axio Vert.A1 microscope or a Keyence BZ-X810 all-in-one fluorescence microscope, and imaged with an exposure time of 2.6 s. Locomotor velocity (µm/s) was determined by measurement of length of trajectories of the *S*Tm movement.

### Invasion assay into HeLa and Caco-2 cells

The assay for invasion of HeLa cells was performed as previously described [69]. We alternatively used Caco-2 cells as well. *S*Tm grown in LB to the late logarithmic phase was resuspended in binding buffer (25 mM MES [pH 6.0], 25 mM NaCl) and incubated with 10 µM RegIIIβ for 15 min at 37°C. Bacteria were added to HeLa cells or Caco-2 cells at a multiplicity of infection (MOI) of 10 and incubated for 3 h or 1 h at 37°C with 5% CO_2_. Extracellular bacteria were killed by adding 100 µg/ml gentamicin and incubating for 1 h at 37°C with 5% CO_2_. Cells were then washed with PBS and lysed with 1% Triton X-100. Serial dilutions were plated on LB agar plates to determine the number of cell-invaded bacteria (intracellular bacteria). The number of input bacteria was defined as 100%.

### Replication assay within HeLa cells

*S*Tm grown in LB to the late logarithmic growth phase was resuspended in the binding buffer (25 mM MES [pH 6.0], 25 mM NaCl), and incubate with 10 µM RegIIIβ for 15 min at 37°C. Bacteria were infected into HeLa cells at an MOI of 10 for 3 h at 37°C with 5% CO_2_. The cells were washed with PBS, incubated with cell culture medium containing 50 µg/ml gentamicin for 17 h at 37°C with 5% CO_2_. After washing with PBS, the cells were lysed with 1% Triton X-100. To determine the numbers of intracellular replicated bacteria, the serial diluted samples were plated on LB agar plates.

### RNA isolation from bacteria and reverse transcription quantitative real-time PCR

Bacteria grown in LB until the logarithmic growth phase were isolated from the medium by centrifugation, and RNA was isolated using a Direct-zol RNA MiniPrep kit (Zymo Research) following the manufacturer’s protocol. RNA concentrations and purity were determined spectrophotometrically using a NanoDrop One Spectrophotometer (Thermo Fisher Scientific). The extracted RNA was also verified by PCR to confirm the absence of contaminating DNA. Reverse transcription was performed using TaqMan Reverse Transcription reagents (Invitrogen). Quantitative real-time PCR (qPCR) was performed using SYBR Fast qPCR master mix (Kapa Biosystems) on CFX Opus 96 real-time PCR detection system (Bio-Rad) to amplify the target genes with specific primer pairs listed in S3 Table. Relative transcript levels were normalized to the *rpoD* gene and calculated by using the 2^-∆*CT*^ method [34].

### Flagellin production and transport

Logarithmically grown bacteria were diluted to OD_600_ of 0.8 in LB medium. A volume of 1 ml was centrifuged, and the pellet was resuspended in 1 ml of binding buffer (25 mM MES [pH 6.0], 25 mM NaCl). A 500 µl of aliquots were incubated with 10 µM RegIIIβ along shaking in a thermomixer for 15 min at 37°C. After the incubation, the samples were vortexed at high speed for 5 min to shear the flagellin from the bacterial surface into the supernatant [70]. The pellet was resolved in SDS-PAGE sample buffer (lysate). The supernatant was transferred into a fresh tube, and subjected to trichloroacetic acid (TCA) precipitation at a final concentration of 10%. After the incubation on ice for 15 min, the TCA-precipitated proteins were harvested by centrifugation at 4°C for 10 min at 16,000 × *g*, followed by washing with cold acetone. The resulting pellet was resuspended in SDS-PAGE sample buffer (sheared). Lysate (intracellular) and sheared (transported) proteins were analyzed by SDS-PAGE and Western blotting using anti-*Salmonella* type H-i serum (Denka Seiken Co., Ltd.).

### Membrane potential assay using DiSC_3_(5)

Bacteria were grown in LB until the logarithmic growth phase, collected by centrifugation. The pellet was washed with binding buffer (25 mM MES [pH 6.0], 25 mM NaCl) or LB, resuspended in binding buffer or LB. The membrane potential sensitive dye 3,3’-Dipropylthiadicarbocyanine iodide (DiSC_3_(5)) [53] was added at final concentration of 1 µM. The samples were incubated under shaking conditions in a thermomixer for 15 min at 37°C. After the incubation, the samples were mixed with 10 µM RegIIIβ or 1 mM the ionophore carbonyl cyanide m-chlorophenyl hydrazone (CCCP) for 5 min at 37°C, immediately the fluorescence of the DiSC_3_(5)-intracellular lipid component complex was measured by using Spark^®^ (TECAN) with excitation and emission wavelengths of 620 and 685 nm.

### Determination of intracellular ATP levels

Intracellular ATP concentrations were determined using the CheckLite HS Set (Kikkoman Biochemifa Company) according to the manufacturer’s instructions. Bioluminescence was measured using Spark^®^ (TECAN).

### Statistical analysis

All statistical analyses were conducted using GraphPad Prism 10 for MacOS (GraphPad Software). A one sample *t* test, the Mann-Whitney U test, One-way ANOVA test followed by Dunnett’s multiple comparison test, and an unpaired or paired *t* test were used to assess statistical significance. Used test is described in the figure legends.

## Supporting information

S1 Fig RegIIIβ expression and *S*Tm loads in mouse infection model without streptomycin pretreatment. Naïve C57BL/6 mice were infected with *S*Tm by oral gavage. RegIIIβ in the fecal samples at 24 hours post-infection was analyzed by SDS-PAGE and Western blotting using anti- *RegIIIβ*^+/-^ mice antibodies (A). Samples 1–3 were obtained from *S*Tm infected *RegIIIβ*^+/+^ or *RegIIIβ*^+/-^ mice, whereas samples 4–6 were obtained from *RegIIIβ*^-/-^ littermates. A control is equivalent to 0.25 µg of RegIIIβ. Mice were sacrificed and *S*Tm loads in the feces (B), mesenteric lymph node (C), spleen (D), and liver (E) were determined. *n* indicates the number of data points. Bars, median with interquartile range. Black dotted line, detection limit. Two-tailed Mann-Whitney U test. *P* > 0.05 not significant (ns).
(TIF)

S2 Fig RegIIIβ binding enhances invasiveness of *S*Tm into HeLa cells. A *S*Tm strain was preincubated with recombinant RegIIIβ and added to monolayer cultures of HeLa cells, followed by a 1-h incubation. Quantified invasiveness was determined by defining the input *S*Tm cells (inoculum) as 100%. *n* indicates the number of data points. Data were obtained from two independent experiments. Bars, median with interquartile range. Two-tailed Mann‒Whitney U test. *P* < 0.01 (^**^).
(TIF)

S3 Fig RegIIIβ preincubation leads to increased invasion of *S*Tm into Caco-2 cells. A *S*Tm strain was preincubated with recombinant RegIIIβ and added to monolayer cultures of Caco-2 cells, followed by a 3-h incubation. Quantified invasiveness was determined by defining the input *S*Tm cells (inoculum) as 100%. *n* indicates the number of data points. Data were obtained from two independent experiments. Bars, median with interquartile range. Two-tailed Mann‒Whitney U test. *P* < 0.05 (^*^).
(TIF)

S4 Fig RegIIIβ binding does not enhance the invasiveness of a nonmotile *S*Tm into HeLa cell. A nonmotile *S*Tm ∆*fliGHI* was preincubated with recombinant RegIIIβ and added the monolayer cultures of HeLa cells. Quantified invasiveness was determined by defining the input *S*Tm cells (inoculum) as 100%. *n* indicates the number of data points. Data were obtained from two independent experiments. Bars, median with interquartile range. Unpaired *t* test. *P* > 0.05 not significant (ns).
(TIF)

S5 Fig RegIIIβ binding enhances the invasiveness of a T3SS-deficient *S*Tm into HeLa cells. A T3SS-deficient *S*Tm (∆*invG* ∆*ssaV*::*cat*) was preincubated with recombinant RegIIIβ and added the monolayer cultures of HeLa cells. If needed, the centrifugation step was applied for close contact between *S*Tm cells and HeLa cells. Quantified invasiveness was determined by defining the input *S*Tm cells (inoculum) as 100%. *n* indicates the number of data points. Data were obtained from two independent experiments. Bars, median with interquartile range. A one-way ANOVA followed by Dunnett’s multiple comparisons test. *P* > 0.05 not significant (ns), *P* < 0.05 (^*^), *P* < 0.01 (^**^), *P* < 0.001 (^***^), *P* < 0.0001 (^****^).
(TIF)

S6 Fig RegIIIβ binding facilitates intracellular replication of *S*Tm within HeLa cells. HeLa cells were infected with *S*Tm cells or RegIIIβ-pretreated *S*Tm cells for 20 h. The ability to replicate within HeLa cells was determined as fold increase (20 h/4 h infection). *n* = 6 for each group (RegIIIβ-pretreated or untreated *S*Tm cells). Bars indicate the median value with interquartile range for each group. Two-tailed Mann‒Whitney U test. *P* > 0.05 not significant (ns), *P* < 0.05 (^*^), *P* < 0.01 (^**^), *P* < 0.001 (^***^), *P* < 0.0001 (^****^).
(TIF)

S7 Fig RegIIIβ kills *E*. *coli* strain LF82. (A) Microscopy images of *E*. *coli* strain LF82 expressing green fluorescent proteins. The bacterial strains were preincubated with recombinant RegIIIβ, placed on a glass slide, sealed under a glass coverslip, and observed by fluorescence microscopy (exposure time: 2.6 s). Red scale bar, 10 µm. Arrowheads indicate aggregated bacterial cells. (B) *In vitro* killing by RegIIIβ. Percentage of CFUs remaining after exposure to recombinant RegIIIβ. *S*Tm strain SL1344 and *E*. *coli* strain LF82 were grown to logarithmic growth phase and incubated with RegIIIβ. After incubation for 30 min at 37°C, viable bacteria were quantified by dilution plating on selective media. *n* = 4. Data are median from two independent experiments. (C) Growth kinetics in the presence of RegIIIβ. Mixture of *S*Tm strain SL1344 and *E*. *coli* strain LF82 was incubated with 10 µM RegIIIβ. Bacterial loads (CFU/ml) of individual strains were determined by selective plating: SL1344 was grown in agar medium containing streptomycin, whereas the agar medium containing ampicillin was used to isolate LF82. *n* indicates the number of data points. Bars, median with interquartile range. Unpaired *t* test. *P* > 0.05 not significant (ns), *P* < 0.05 (^*^), *P* < 0.01 (^**^), *P* < 0.001 (^***^), *P* < 0.0001 (^****^).
(TIF)

S8 Fig RT-qPCR analysis of flagellar genes. (A-C) Transcript levels of flagellar genes (class 1: *flhD* and *flhC*; class 2: *fliA* and *flgB*; class 3: *fliC*, *motB* and *fljB*) relative to *rpoD*. *n* is indicated by the number of dots. Bars, median with interquartile range. Unpaired *t* test. *P* > 0.05 not significant (ns), *P* < 0.05 (^*^), *P* < 0.01 (^**^), *P* < 0.001 (^***^), *P* < 0.0001 (^****^).
(TIF)

S9 Fig RegIIIβ binding in LB medium enhances activities of flagellar motility and cell invasion. (A) Microscopy images of *S*Tm expressing green fluorescent proteins in LB medium. The bacterial strains were preincubated with recombinant RegIIIβ, placed on a glass slide, sealed under a glass coverslip, and observed by fluorescence microscopy (exposure time: 2.6 s). Red scale bar, 10 µm. (B) Microscopy quantification of *S*Tm locomotor velocity of the experiment in panel B. *n* = 808 [RegIIIβ-], and 556 [RegIIIβ+]. Bars, median with interquartile range. Two-tailed Mann‒Whitney U test. *P* > 0.05 not significant (ns), *P* < 0.05 (^*^), *P* < 0.01 (^**^), *P* < 0.001 (^***^), *P* < 0.0001 (^****^). (C) *S*Tm WT was preincubated with recombinant RegIIIβ in LB medium and added the monolayer cultures of HeLa cells, followed by centrifugation for close contact between *S*Tm cells and HeLa cells. Quantified invasiveness was determined by defining the input *S*Tm cells (inoculum) as 100%. *n* indicates the number of data points. Data were obtained from two independent experiments. Bars, median with interquartile range. A one-way ANOVA followed by Dunnett’s multiple comparisons test. *P* > 0.05 not significant (ns), *P* < 0.05 (^*^), *P* < 0.01 (^**^), *P* < 0.001 (^***^), *P* < 0.0001 (^****^).
(TIF)

S10 Fig RegIIIβ-enhanced flagellar motility plays a critical role in early-stage gut colonization by *S*Tm. C57BL/6 mice were pre-treated with 25 mg of streptomycin by oral gavage 24 h before oral infection with *S*Tm (1:1 mixture of strain 1-WT and strain 2-∆*fliGHI*). Mice were euthanized on day 1 post-infection, and feces were collected. The CI of *S*Tm loads recovered from the feces was determined by selective plating. Bars, median with interquartile range. Two-tailed Mann‒Whitney U test. *P* > 0.05 not significant (ns), *P* < 0.05 (^*^), *P* < 0.01 (^**^), *P* < 0.001 (^***^), *P* < 0.0001 (^****^).
(TIF)

S1 Table Results of peptide matches using Mascot software. (DOCX)

S2 Table Bacterial strains and plasmids used in this study. (DOCX)

S3 Table Oligonucleotide primers in this study. (DOCX)

S1 Raw Data Excel spreadsheet containing, in separate sheets for each figure, the underlying and individual numerical data for Figs 1C and 1D, 2B–2H, 3B–3F, 4B–4F, S1B–S1E, S2–S6, S7B and S7C, S8A–S8C, S9B and S9C, S10. (XLSX)

S1 Raw Images Uncropped pictures used in Figs 1B, 1F, 2E, 3A and S1A. (PDF)

## References

[1] Yang D, Chertov O, Bykovskaia SN, Chen Q, Buffo MJ, Shogan J, et al. Beta-defensins: linking innate and adaptive immunity through dendritic and T cell CCR6. Science. 1999;286(5439):525–8. doi: 10.1126/science.286.5439.525 10521347

[2] Wu Z, Hoover DM, Yang D, Boulègue C, Santamaria F, Oppenheim JJ, et al. Engineering disulfide bridges to dissect antimicrobial and chemotactic activities of human beta-defensin 3. Proc Natl Acad Sci U S A. 2003;100(15):8880–5. doi: 10.1073/pnas.1533186100 12840147 PMC166407

[3] Röhrl J, Yang D, Oppenheim JJ, Hehlgans T. Identification and Biological Characterization of Mouse beta-defensin 14, the orthologue of human beta-defensin 3. J Biol Chem. 2008;283(9):5414–9. doi: 10.1074/jbc.M709103200 18167348

[4] Vongsa RA, Zimmerman NP, Dwinell MB. CCR6 regulation of the actin cytoskeleton orchestrates human beta defensin-2- and CCL20-mediated restitution of colonic epithelial cells. J Biol Chem. 2009;284(15):10034–45. doi: 10.1074/jbc.M805289200 19233848 PMC2665058

[5] Otte J-M, Werner I, Brand S, Chromik AM, Schmitz F, Kleine M, et al. Human beta defensin 2 promotes intestinal wound healing in vitro. J Cell Biochem. 2008;104(6):2286–97. doi: 10.1002/jcb.21787 18449938

[6] Narushima Y, Unno M, Nakagawara K, Mori M, Miyashita H, Suzuki Y, et al. Structure, chromosomal localization and expression of mouse genes encoding type III Reg, RegIII alpha, RegIII beta, RegIII gamma. Gene. 1997;185(2):159–68. doi: 10.1016/s0378-1119(96)00589-6 9055810

[7] Stelter C, Käppeli R, König C, Krah A, Hardt W-D, Stecher B, et al. Salmonella-induced mucosal lectin RegIIIβ kills competing gut microbiota. PLoS One. 2011;6(6):e20749. doi: 10.1371/journal.pone.0020749 21694778 PMC3111430

[8] Miki T, Hardt W-D. Outer membrane permeabilization is an essential step in the killing of gram-negative bacteria by the lectin RegIIIβ. PLoS One. 2013;8(7):e69901. doi: 10.1371/journal.pone.0069901 23922847 PMC3726741

[9] Miki T, Holst O, Hardt W-D. The bactericidal activity of the C-type lectin RegIIIβ against Gram-negative bacteria involves binding to lipid A. J Biol Chem. 2012;287(41):34844–55. doi: 10.1074/jbc.M112.399998 22896700 PMC3464586

[10] Cash HL, Whitham CV, Behrendt CL, Hooper LV. Symbiotic bacteria direct expression of an intestinal bactericidal lectin. Science. 2006;313(5790):1126–30. doi: 10.1126/science.1127119 16931762 PMC2716667

[11] Vaishnava S, Behrendt CL, Ismail AS, Eckmann L, Hooper LV. Paneth cells directly sense gut commensals and maintain homeostasis at the intestinal host-microbial interface. Proc Natl Acad Sci U S A. 2008;105(52):20858–63. doi: 10.1073/pnas.0808723105 19075245 PMC2603261

[12] Ogawa H, Fukushima K, Naito H, Funayama Y, Unno M, Takahashi K, et al. Increased expression of HIP/PAP and regenerating gene III in human inflammatory bowel disease and a murine bacterial reconstitution model. Inflamm Bowel Dis. 2003;9(3):162–70. doi: 10.1097/00054725-200305000-00003 12792221

[13] Brandl K, Plitas G, Schnabl B, DeMatteo RP, Pamer EG. MyD88-mediated signals induce the bactericidal lectin RegIII gamma and protect mice against intestinal Listeria monocytogenes infection. J Exp Med. 2007;204(8):1891–900. doi: 10.1084/jem.20070563 17635956 PMC2118673

[14] Brandl K, Plitas G, Mihu CN, Ubeda C, Jia T, Fleisher M, et al. Vancomycin-resistant enterococci exploit antibiotic-induced innate immune deficits. Nature. 2008;455(7214):804–7. doi: 10.1038/nature07250 18724361 PMC2663337

[15] Natividad JMM, Petit V, Huang X, de Palma G, Jury J, Sanz Y, et al. Commensal and probiotic bacteria influence intestinal barrier function and susceptibility to colitis in Nod1-/-; Nod2-/- mice. Inflamm Bowel Dis. 2012;18(8):1434–46. doi: 10.1002/ibd.22848 22162005

[16] Liang SC, Tan X-Y, Luxenberg DP, Karim R, Dunussi-Joannopoulos K, Collins M, et al. Interleukin (IL)-22 and IL-17 are coexpressed by Th17 cells and cooperatively enhance expression of antimicrobial peptides. J Exp Med. 2006;203(10):2271–9. doi: 10.1084/jem.20061308 16982811 PMC2118116

[17] Zheng Y, Valdez PA, Danilenko DM, Hu Y, Sa SM, Gong Q, et al. Interleukin-22 mediates early host defense against attaching and effacing bacterial pathogens. Nat Med. 2008;14(3):282–9. doi: 10.1038/nm1720 18264109

[18] Kinnebrew MA, Ubeda C, Zenewicz LA, Smith N, Flavell RA, Pamer EG. Bacterial flagellin stimulates Toll-like receptor 5-dependent defense against vancomycin-resistant Enterococcus infection. J Infect Dis. 2010;201(4):534–43. doi: 10.1086/650203 20064069 PMC2811237

[19] Geddes K, Rubino SJ, Magalhaes JG, Streutker C, Le Bourhis L, Cho JH, et al. Identification of an innate T helper type 17 response to intestinal bacterial pathogens. Nat Med. 2011;17(7):837–44. doi: 10.1038/nm.2391 21666695

[20] van Ampting MTJ, Loonen LMP, Schonewille AJ, Konings I, Vink C, Iovanna J, et al. Intestinally secreted C-type lectin Reg3b attenuates salmonellosis but not listeriosis in mice. Infect Immun. 2012;80(3):1115–20. doi: 10.1128/IAI.06165-11 22252863 PMC3294648

[21] van Ampting MTJ, Rodenburg W, Vink C, Kramer E, Schonewille AJ, Keijer J, et al. Ileal mucosal and fecal pancreatitis associated protein levels reflect severity of salmonella infection in rats. Dig Dis Sci. 2009;54(12):2588–97. doi: 10.1007/s10620-008-0685-0 19160051

[22] Loonen LMP, Stolte EH, Jaklofsky MTJ, Meijerink M, Dekker J, van Baarlen P, et al. REG3γ-deficient mice have altered mucus distribution and increased mucosal inflammatory responses to the microbiota and enteric pathogens in the ileum. Mucosal Immunol. 2014;7(4):939–47. doi: 10.1038/mi.2013.109 24345802

[23] Devleesschauwer B, Haagsma JA, Angulo FJ, Bellinger DC, Cole D, Döpfer D, et al. Methodological Framework for World Health Organization Estimates of the Global Burden of Foodborne Disease. PLoS One. 2015;10(12):e0142498. doi: 10.1371/journal.pone.0142498 26633883 PMC4668830

[24] Kirk MD, Pires SM, Black RE, Caipo M, Crump JA, Devleesschauwer B, et al. World Health Organization Estimates of the Global and Regional Disease Burden of 22 Foodborne Bacterial, Protozoal, and Viral Diseases, 2010: A Data Synthesis. PLoS Med. 2015;12(12):e1001921. doi: 10.1371/journal.pmed.1001921 26633831 PMC4668831

[25] DuPont HL. Persistent Diarrhea: A Clinical Review. JAMA. 2016;315(24):2712–23. doi: 10.1001/jama.2016.7833 27357241

[26] Lanata CF, Fischer-Walker CL, Olascoaga AC, Torres CX, Aryee MJ, Black RE, et al. Global causes of diarrheal disease mortality in children <5 years of age: a systematic review. PLoS One. 2013;8(9):e72788. doi: 10.1371/journal.pone.0072788 24023773 PMC3762858

[27] Besser JM. Salmonella epidemiology: A whirlwind of change. Food Microbiol. 2018;71:55–9. doi: 10.1016/j.fm.2017.08.018 29366469

[28] Misselwitz B, Barrett N, Kreibich S, Vonaesch P, Andritschke D, Rout S, et al. Near surface swimming of Salmonella Typhimurium explains target-site selection and cooperative invasion. PLoS Pathog. 2012;8(7):e1002810. doi: 10.1371/journal.ppat.1002810 22911370 PMC3406100

[29] Stecher B, Hapfelmeier S, Müller C, Kremer M, Stallmach T, Hardt W-D. Flagella and chemotaxis are required for efficient induction of Salmonella enterica serovar Typhimurium colitis in streptomycin-pretreated mice. Infect Immun. 2004;72(7):4138–50. doi: 10.1128/IAI.72.7.4138-4150.2004 15213159 PMC427403

[30] Galán JE, Curtiss R 3rd. Cloning and molecular characterization of genes whose products allow Salmonella typhimurium to penetrate tissue culture cells. Proc Natl Acad Sci U S A. 1989;86(16):6383–7. doi: 10.1073/pnas.86.16.6383 2548211 PMC297844

[31] Hapfelmeier S, Stecher B, Barthel M, Kremer M, Müller AJ, Heikenwalder M, et al. The Salmonella pathogenicity island (SPI)-2 and SPI-1 type III secretion systems allow Salmonella serovar typhimurium to trigger colitis via MyD88-dependent and MyD88-independent mechanisms. J Immunol. 2005;174(3):1675–85. doi: 10.4049/jimmunol.174.3.1675 15661931

[32] Watson PR, Paulin SM, Bland AP, Jones PW, Wallis TS. Characterization of intestinal invasion by Salmonella typhimurium and Salmonella dublin and effect of a mutation in the invH gene. Infect Immun. 1995;63(7):2743–54. doi: 10.1128/iai.63.7.2743-2754.1995 7790093 PMC173367

[33] Stecher B, Robbiani R, Walker AW, Westendorf AM, Barthel M, Kremer M, et al. Salmonella enterica serovar typhimurium exploits inflammation to compete with the intestinal microbiota. PLoS Biol. 2007;5(10):2177–89. doi: 10.1371/journal.pbio.0050244 17760501 PMC1951780

[34] Litvak Y, Mon KKZ, Nguyen H, Chanthavixay G, Liou M, Velazquez EM, et al. Commensal Enterobacteriaceae Protect against Salmonella Colonization through Oxygen Competition. Cell Host Microbe. 2019;25(1):128-139.e5. doi: 10.1016/j.chom.2018.12.003 30629913 PMC12036633

[35] Rivera-Chávez F, Lopez CA, Zhang LF, García-Pastor L, Chávez-Arroyo A, Lokken KL, et al. Energy Taxis toward Host-Derived Nitrate Supports a Salmonella Pathogenicity Island 1-Independent Mechanism of Invasion. mBio. 2016;7(4):e00960-16. doi: 10.1128/mBio.00960-16 27435462 PMC4958259

[36] Winter SE, Thiennimitr P, Winter MG, Butler BP, Huseby DL, Crawford RW, et al. Gut inflammation provides a respiratory electron acceptor for Salmonella. Nature. 2010;467(7314):426–9. doi: 10.1038/nature09415 20864996 PMC2946174

[37] Maier L, Diard M, Sellin ME, Chouffane E-S, Trautwein-Weidner K, Periaswamy B, et al. Granulocytes impose a tight bottleneck upon the gut luminal pathogen population during Salmonella typhimurium colitis. PLoS Pathog. 2014;10(12):e1004557. doi: 10.1371/journal.ppat.1004557 25522364 PMC4270771

[38] Barthel M, Hapfelmeier S, Quintanilla-Martínez L, Kremer M, Rohde M, Hogardt M, et al. Pretreatment of mice with streptomycin provides a Salmonella enterica serovar Typhimurium colitis model that allows analysis of both pathogen and host. Infect Immun. 2003;71(5):2839–58. doi: 10.1128/IAI.71.5.2839-2858.2003 12704158 PMC153285

[39] Kaiser P, Diard M, Stecher B, Hardt W-D. The streptomycin mouse model for Salmonella diarrhea: functional analysis of the microbiota, the pathogen’s virulence factors, and the host’s mucosal immune response. Immunol Rev. 2012;245(1):56–83. doi: 10.1111/j.1600-065X.2011.01070.x 22168414

[40] Miki T, Goto R, Fujimoto M, Okada N, Hardt W-D. The Bactericidal Lectin RegIIIβ Prolongs Gut Colonization and Enteropathy in the Streptomycin Mouse Model for Salmonella Diarrhea. Cell Host Microbe. 2017;21(2):195–207. doi: 10.1016/j.chom.2016.12.008 28111202

[41] Kreuzer M, Hardt W-D. How Food Affects Colonization Resistance Against Enteropathogenic Bacteria. Annu Rev Microbiol. 2020;74:787–813. doi: 10.1146/annurev-micro-020420-013457 32692613

[42] Mukherjee S, Partch CL, Lehotzky RE, Whitham CV, Chu H, Bevins CL, et al. Regulation of C-type lectin antimicrobial activity by a flexible N-terminal prosegment. J Biol Chem. 2009;284(8):4881–8. doi: 10.1074/jbc.M808077200 19095652 PMC2643518

[43] Hume PJ, Singh V, Davidson AC, Koronakis V. Swiss Army Pathogen: The Salmonella Entry Toolkit. Front Cell Infect Microbiol. 2017;7:348. doi: 10.3389/fcimb.2017.00348 28848711 PMC5552672

[44] Rosselin M, Virlogeux-Payant I, Roy C, Bottreau E, Sizaret P-Y, Mijouin L, et al. Rck of Salmonella enterica, subspecies enterica serovar enteritidis, mediates zipper-like internalization. Cell Res. 2010;20(6):647–64. doi: 10.1038/cr.2010.45 20368731

[45] Lambert MA, Smith SGJ. The PagN protein of Salmonella enterica serovar Typhimurium is an adhesin and invasin. BMC Microbiol. 2008;8:142. doi: 10.1186/1471-2180-8-142 18778463 PMC2553418

[46] Lorkowski M, Felipe-López A, Danzer CA, Hansmeier N, Hensel M. Salmonella enterica invasion of polarized epithelial cells is a highly cooperative effort. Infect Immun. 2014;82(6):2657–67. doi: 10.1128/IAI.00023-14 24711567 PMC4019164

[47] Darfeuille-Michaud A, Neut C, Barnich N, Lederman E, Di Martino P, Desreumaux P, et al. Presence of adherent Escherichia coli strains in ileal mucosa of patients with Crohn’s disease. Gastroenterology. 1998;115(6):1405–13. doi: 10.1016/s0016-5085(98)70019-8 9834268

[48] Boudeau J, Glasser AL, Masseret E, Joly B, Darfeuille-Michaud A. Invasive ability of an Escherichia coli strain isolated from the ileal mucosa of a patient with Crohn’s disease. Infect Immun. 1999;67(9):4499–509. doi: 10.1128/IAI.67.9.4499-4509.1999 10456892 PMC96770

[49] Barnich N, Boudeau J, Claret L, Darfeuille-Michaud A. Regulatory and functional co-operation of flagella and type 1 pili in adhesive and invasive abilities of AIEC strain LF82 isolated from a patient with Crohn’s disease. Mol Microbiol. 2003;48(3):781–94. doi: 10.1046/j.1365-2958.2003.03468.x 12694621

[50] Carvalho FA, Barnich N, Sivignon A, Darcha C, Chan CHF, Stanners CP, et al. Crohn’s disease adherent-invasive Escherichia coli colonize and induce strong gut inflammation in transgenic mice expressing human CEACAM. J Exp Med. 2009;206(10):2179–89. doi: 10.1084/jem.20090741 19737864 PMC2757893

[51] Erhardt M, Mertens ME, Fabiani FD, Hughes KT. ATPase-independent type-III protein secretion in Salmonella enterica. PLoS Genet. 2014;10(11):e1004800. doi: 10.1371/journal.pgen.1004800 25393010 PMC4230889

[52] Gabel CV, Berg HC. The speed of the flagellar rotary motor of Escherichia coli varies linearly with protonmotive force. Proc Natl Acad Sci U S A. 2003;100(15):8748–51. doi: 10.1073/pnas.1533395100 12857945 PMC166384

[53] Buttress JA, Halte M, Te Winkel JD, Erhardt M, Popp PF, Strahl H. A guide for membrane potential measurements in Gram-negative bacteria using voltage-sensitive dyes. Microbiology (Reading). 2022;168(9):10.1099/mic.0.001227. doi: 10.1099/mic.0.001227 36165741

[54] Miki T, Okada N, Hardt W-D. Inflammatory bactericidal lectin RegIIIβ: Friend or foe for the host? Gut Microbes. 2018;9(2):179–87. doi: 10.1080/19490976.2017.1387344 28985140 PMC5989794

[55] Morimoto YV, Minamino T. Architecture and Assembly of the Bacterial Flagellar Motor Complex. Subcell Biochem. 2021;96:297–321. doi: 10.1007/978-3-030-58971-4_8 33252734

[56] Matsura S, Shioi J, Imae Y. Motility in Bacillus subtilis driven by an artificial protonmotive force. FEBS Lett. 1977;82(2):187–90. doi: 10.1016/0014-5793(77)80581-4 410660

[57] Minamino T, Kinoshita M, Morimoto YV, Namba K. Activation mechanism of the bacterial flagellar dual-fuel protein export engine. Biophys Physicobiol. 2022;19:e190046. doi: 10.2142/biophysico.bppb-v19.0046 36567733 PMC9751260

[58] Minamino T, Morimoto YV, Hara N, Aldridge PD, Namba K. The Bacterial Flagellar Type III Export Gate Complex Is a Dual Fuel Engine That Can Use Both H+ and Na+ for Flagellar Protein Export. PLoS Pathog. 2016;12(3):e1005495. doi: 10.1371/journal.ppat.1005495 26943926 PMC4778876

[59] Minamino T, Morimoto YV, Kinoshita M, Namba K. Membrane voltage-dependent activation mechanism of the bacterial flagellar protein export apparatus. Proc Natl Acad Sci U S A. 2021;118(22):e2026587118. doi: 10.1073/pnas.2026587118 34035173 PMC8179193

[60] Hawker PC, McKay JS, Turnberg LA. Electrolyte transport across colonic mucosa from patients with inflammatory bowel disease. Gastroenterology. 1980;79(3):508–11. doi: 10.1016/0016-5085(80)90376-5 7429111

[61] Greig E, Sandle GI. Diarrhea in ulcerative colitis. The role of altered colonic sodium transport. Ann N Y Acad Sci. 2000;915:327–32. doi: 10.1111/j.1749-6632.2000.tb05260.x 11193595

[62] Wilck N, Matus MG, Kearney SM, Olesen SW, Forslund K, Bartolomaeus H, et al. Salt-responsive gut commensal modulates TH17 axis and disease. Nature. 2017;551(7682):585–9. doi: 10.1038/nature24628 29143823 PMC6070150

[63] Monteleone I, Marafini I, Dinallo V, Di Fusco D, Troncone E, Zorzi F, et al. Sodium chloride-enriched Diet Enhanced Inflammatory Cytokine Production and Exacerbated Experimental Colitis in Mice. J Crohns Colitis. 2017;11(2):237–45. doi: 10.1093/ecco-jcc/jjw139 27473029

[64] Minamino T, Kinoshita M, Morimoto YV, Namba K. The FlgN chaperone activates the Na+-driven engine of the Salmonella flagellar protein export apparatus. Commun Biol. 2021;4(1):335. doi: 10.1038/s42003-021-01865-0 33712678 PMC7955116

[65] Chaban B, Hughes HV, Beeby M. The flagellum in bacterial pathogens: For motility and a whole lot more. Semin Cell Dev Biol. 2015;46:91–103. doi: 10.1016/j.semcdb.2015.10.032 26541483

[66] Colin R, Ni B, Laganenka L, Sourjik V. Multiple functions of flagellar motility and chemotaxis in bacterial physiology. FEMS Microbiol Rev. 2021;45(6):fuab038. doi: 10.1093/femsre/fuab038 34227665 PMC8632791

[67] Miki T, Kurihara S, Uemura T, Ami Y, Ito M, Haneda T, et al. Intestinal luminal polyamines support the gut colonization of enteric bacterial pathogens by modulating flagellar motility and nitrate respiration. mBio. 2025;16(9):e0178625. doi: 10.1128/mbio.01786-25 40787976 PMC12421874

[68] Hoshino Y, Sakamoto T, Sudo N, Ito M, Haneda T, Okada N, et al. Fatty Acid Homeostasis Tunes Flagellar Motility by Activating Phase 2 Flagellin Expression, Contributing to Salmonella Gut Colonization. Infect Immun. 2022;90(7):e0018422. doi: 10.1128/iai.00184-22 35652649 PMC9302153

[69] Miki T, Hoshino Y, Sudo N, Ito M, Haneda T, Okada N. uvrY Deletion and Acetate Reduce Gut Colonization of Crohn’s Disease-Associated Adherent-Invasive Escherichia coli by Decreasing Expression of Type 1 Fimbriae. Infect Immun. 2022;90(3):e0066221. doi: 10.1128/iai.00662-21 34978926 PMC8929387

[70] Guard-Petter J. Induction of flagellation and a novel agar-penetrating flagellar structure in Salmonella enterica grown on solid media: possible consequences for serological identification. FEMS Microbiol Lett. 1997;149(2):173–80. doi: 10.1111/j.1574-6968.1997.tb10325.x 9141658

