# Supplementary figures and images for: RegIIIβ promotes Salmonella Typhimurium colonization of the gut in the early-stage gastrointestinal infection by enhancing flagella-driven locomotion
Source: PLoS Pathog. 2025 Nov 3;21(11):e1013665. doi: 10.1371/journal.ppat.1013665 (PMC12591440; doi:10.1371/journal.ppat.1013665)

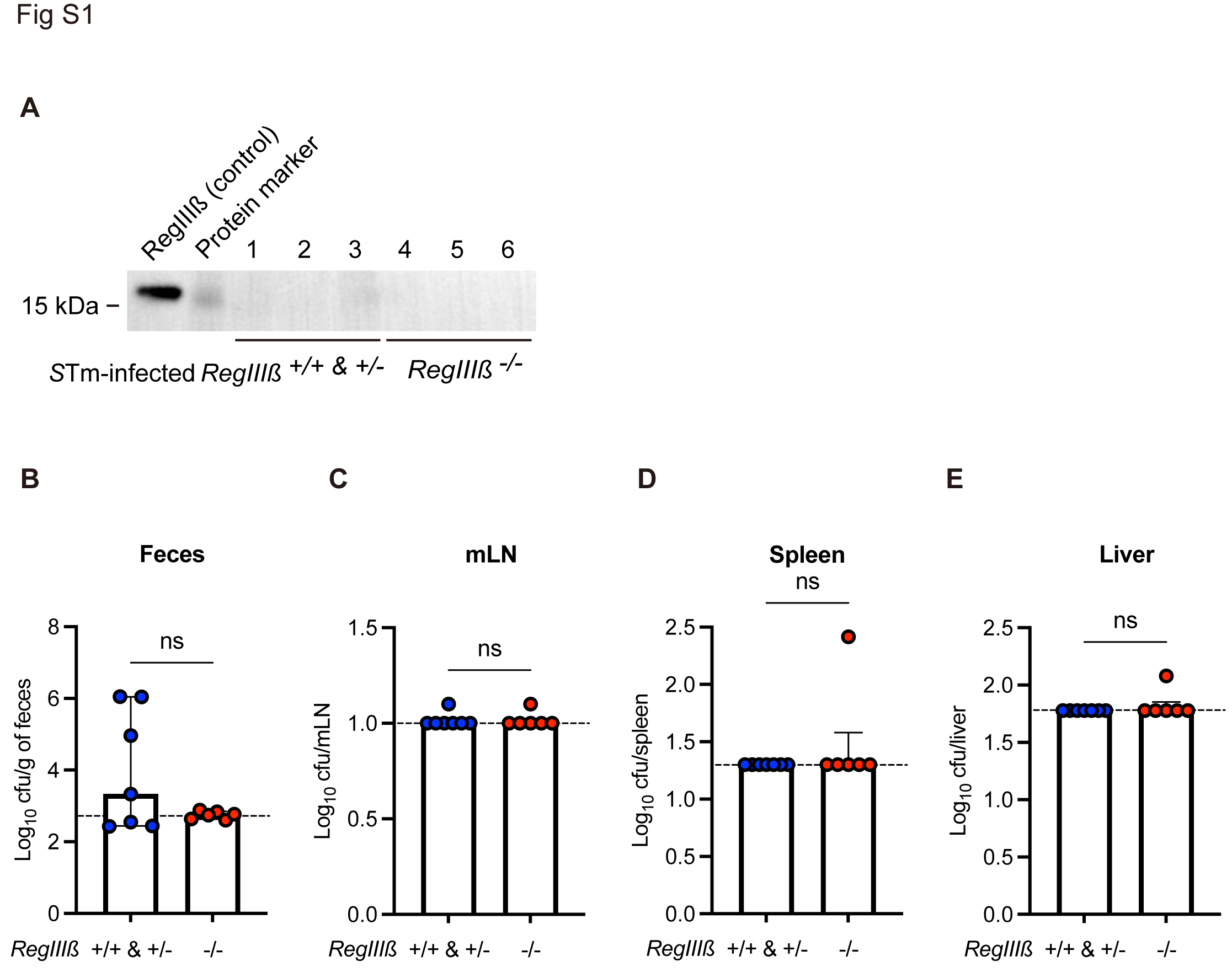

Supplement: S1 Fig — Naïve C57BL/6 mice were infected with STm by oral gavage. RegIIIβ in the fecal samples at 24 hours post-infection was analyzed by SDS-PAGE and Western blotting using anti- RegIIIβ+/- mice antibodies (A). Samples 1–3 were obtained from STm infected RegIIIβ+/+ or RegIIIβ+/- mice, whereas samples 4–6 were obtained from RegIIIβ-/- littermates. A control is equivalent to 0.25 µg of RegIIIβ. Mice were sacrificed and STm loads in the feces (B), mesenteric lymph node (C), spleen (D), and liver (E) were determined. n indicates the number of data points. Bars, median with interquartile range. Black dotted line, detection limit. Two-tailed Mann-Whitney U test. P > 0.05 not significant (ns). (TIF) [file ppat.1013665.s001.tif]

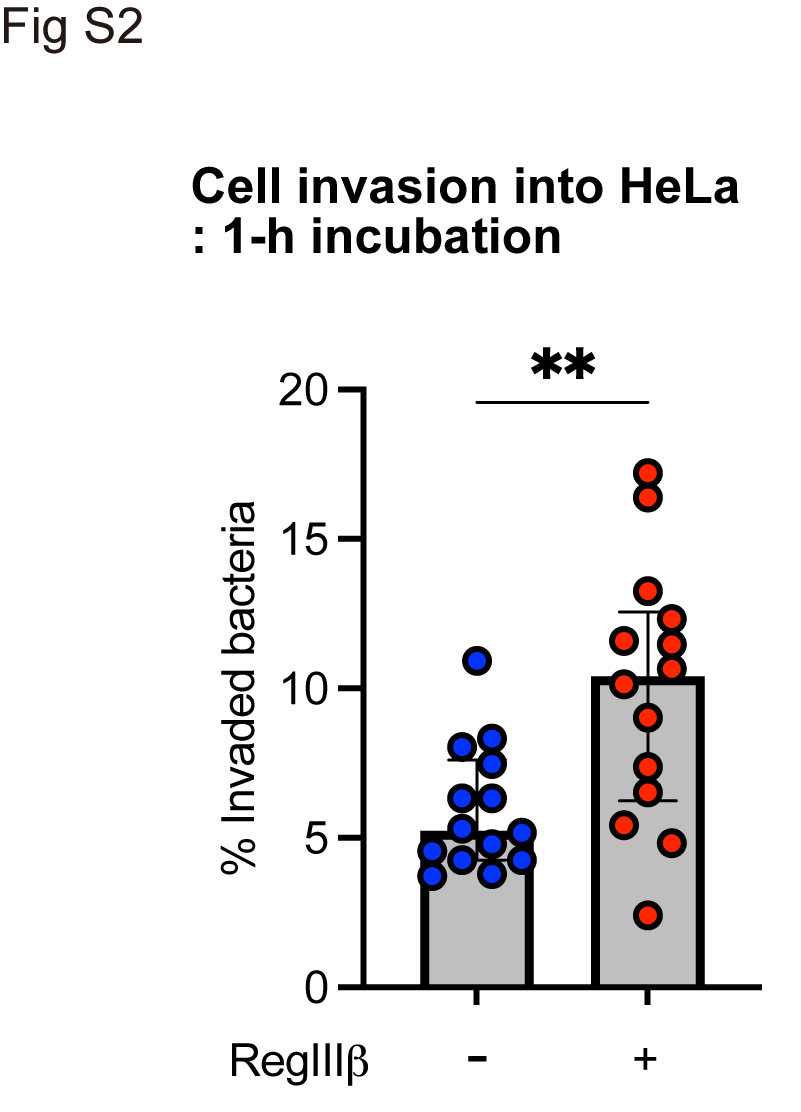

Supplement: S2 Fig — A STm strain was preincubated with recombinant RegIIIβ and added to monolayer cultures of HeLa cells, followed by a 1-h incubation. Quantified invasiveness was determined by defining the input STm cells (inoculum) as 100%. n indicates the number of data points. Data were obtained from two independent experiments. Bars, median with interquartile range. Two-tailed Mann‒Whitney U test. P < 0.01 (**). (TIF) [file ppat.1013665.s002.tif]

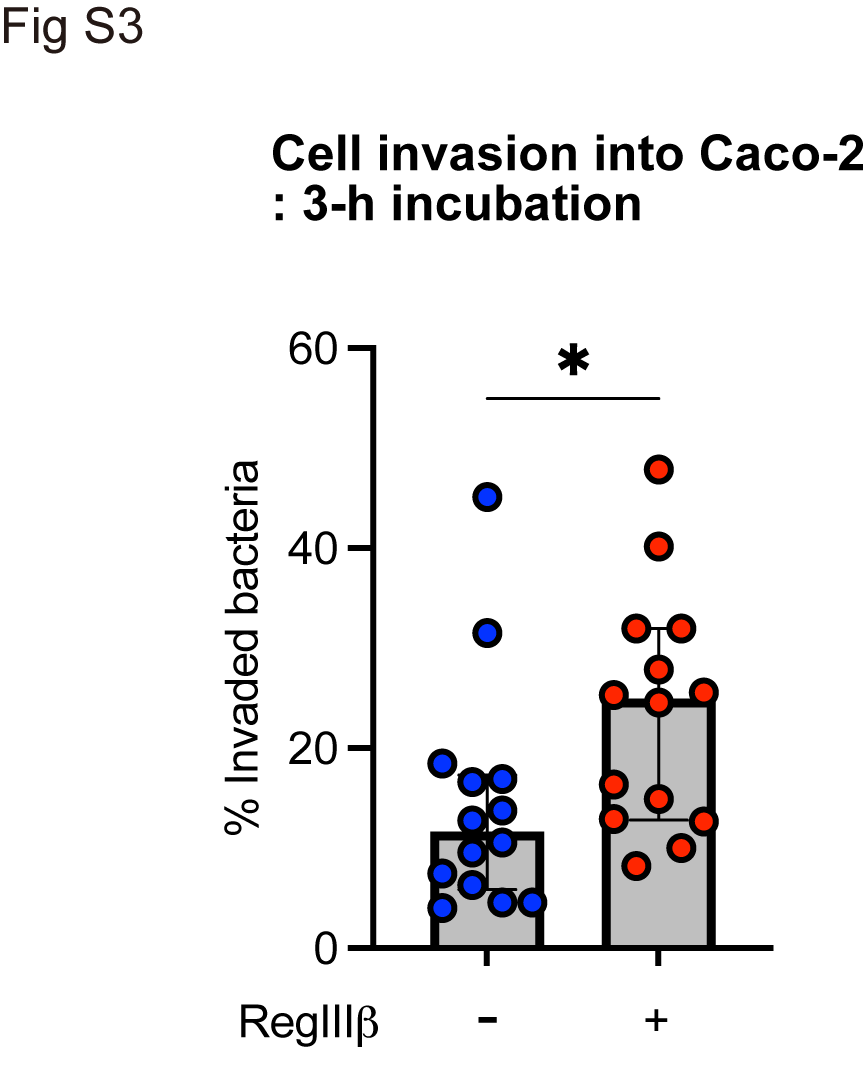

Supplement: S3 Fig — A STm strain was preincubated with recombinant RegIIIβ and added to monolayer cultures of Caco-2 cells, followed by a 3-h incubation. Quantified invasiveness was determined by defining the input STm cells (inoculum) as 100%. n indicates the number of data points. Data were obtained from two independent experiments. Bars, median with interquartile range. Two-tailed Mann‒Whitney U test. P < 0.05 (*). (TIF) [file ppat.1013665.s003.tif]

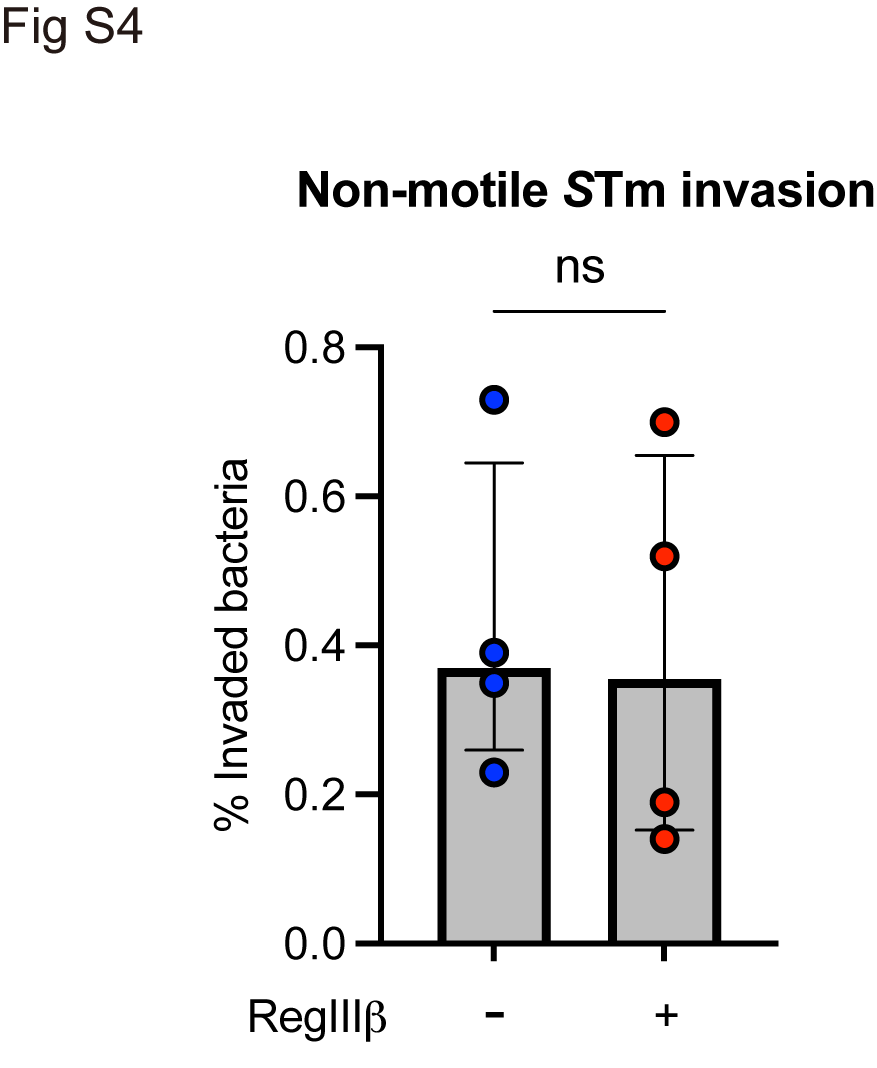

Supplement: S4 Fig — A nonmotile STm ∆fliGHI was preincubated with recombinant RegIIIβ and added the monolayer cultures of HeLa cells. Quantified invasiveness was determined by defining the input STm cells (inoculum) as 100%. n indicates the number of data points. Data were obtained from two independent experiments. Bars, median with interquartile range. Unpaired t test. P > 0.05 not significant (ns). (TIF) [file ppat.1013665.s004.tif]

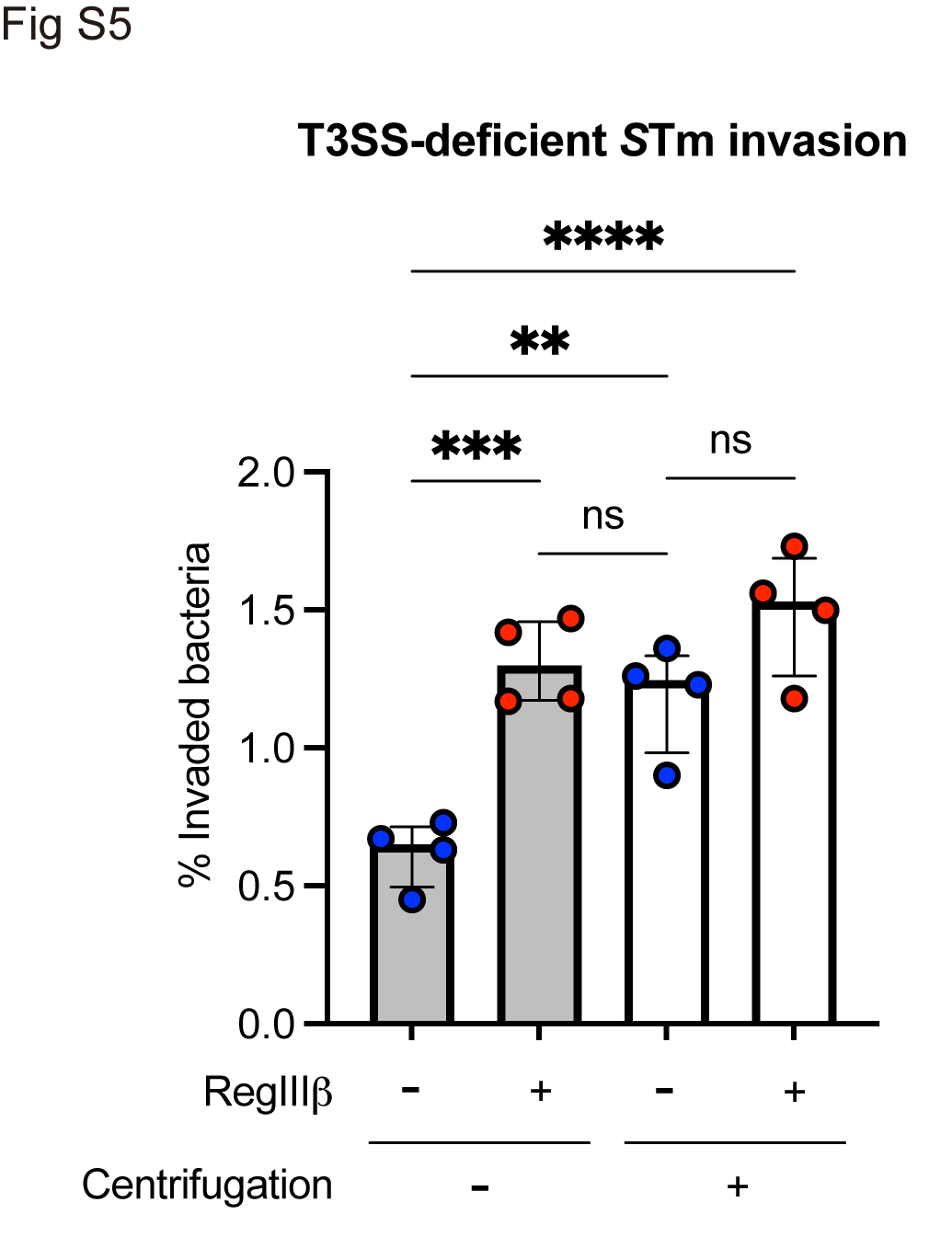

Supplement: S5 Fig — A T3SS-deficient STm (∆invG ∆ssaV::cat) was preincubated with recombinant RegIIIβ and added the monolayer cultures of HeLa cells. If needed, the centrifugation step was applied for close contact between STm cells and HeLa cells. Quantified invasiveness was determined by defining the input STm cells (inoculum) as 100%. n indicates the number of data points. Data were obtained from two independent experiments. Bars, median with interquartile range. A one-way ANOVA followed by Dunnett’s multiple comparisons test. P > 0.05 not significant (ns), P < 0.05 (*), P < 0.01 (**), P < 0.001 (***), P < 0.0001 (****). (TIF) [file ppat.1013665.s005.tif]

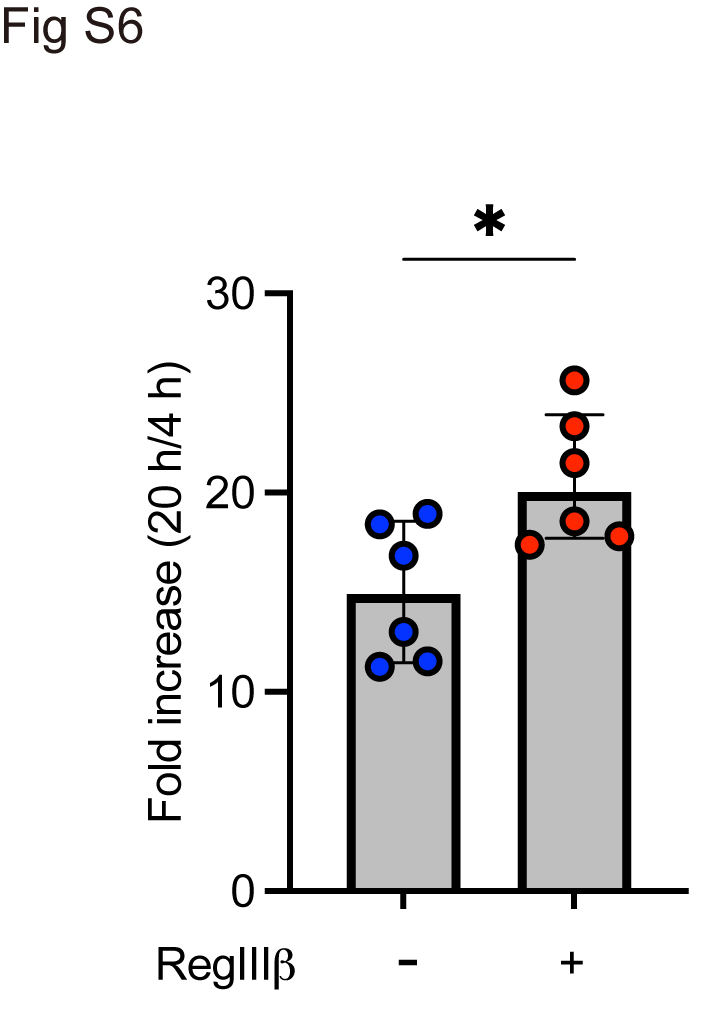

Supplement: S6 Fig — HeLa cells were infected with STm cells or RegIIIβ-pretreated STm cells for 20 h. The ability to replicate within HeLa cells was determined as fold increase (20 h/4 h infection). n = 6 for each group (RegIIIβ-pretreated or untreated STm cells). Bars indicate the median value with interquartile range for each group. Two-tailed Mann‒Whitney U test. P > 0.05 not significant (ns), P < 0.05 (*), P < 0.01 (**), P < 0.001 (***), P < 0.0001 (****). (TIF) [file ppat.1013665.s006.tif]

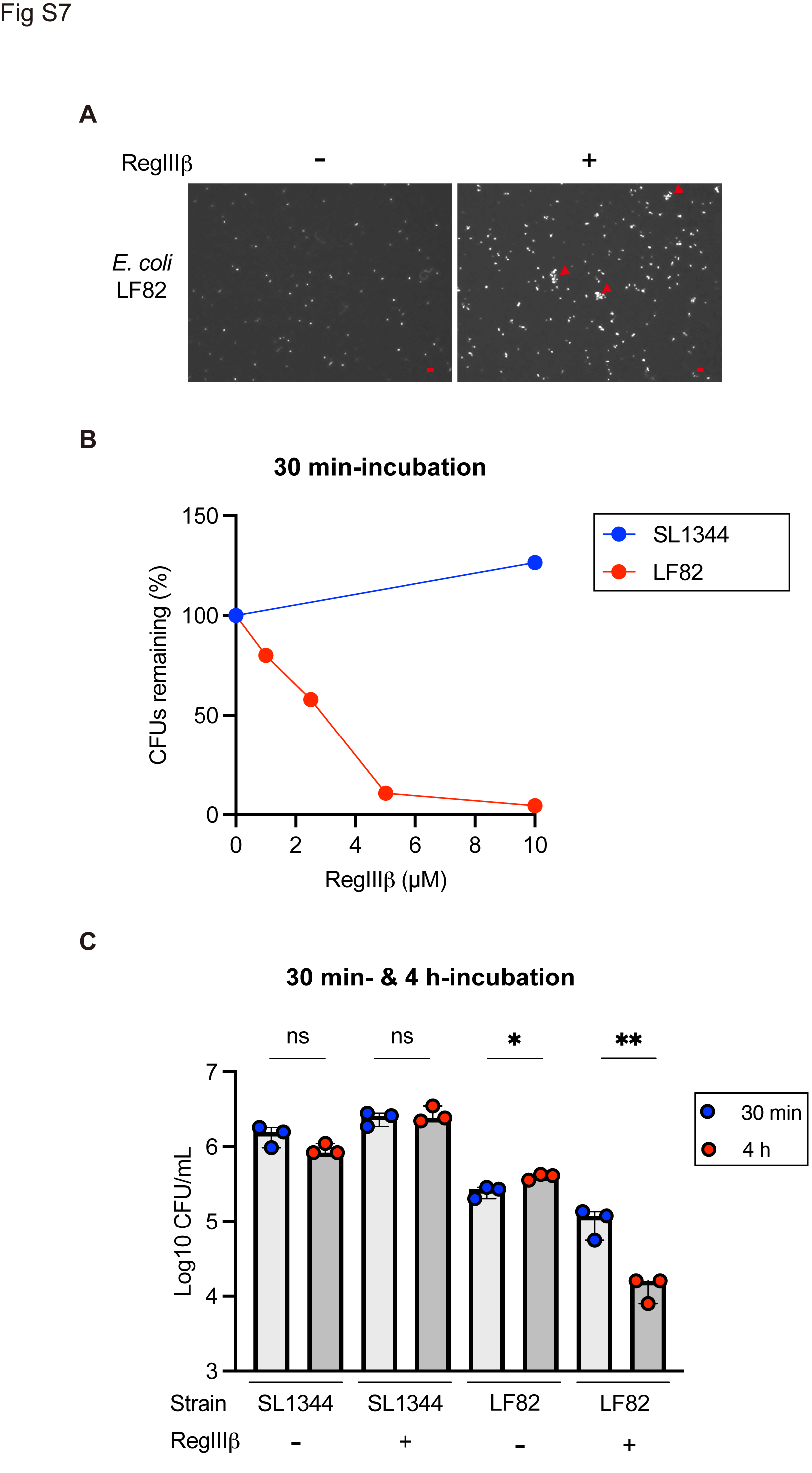

Supplement: S7 Fig — (A) Microscopy images of E. coli strain LF82 expressing green fluorescent proteins. The bacterial strains were preincubated with recombinant RegIIIβ, placed on a glass slide, sealed under a glass coverslip, and observed by fluorescence microscopy (exposure time: 2.6 s). Red scale bar, 10 µm. Arrowheads indicate aggregated bacterial cells. (B) In vitro killing by RegIIIβ. Percentage of CFUs remaining after exposure to recombinant RegIIIβ. STm strain SL1344 and E. coli strain LF82 were grown to logarithmic growth phase and incubated with RegIIIβ. After incubation for 30 min at 37°C, viable bacteria were quantified by dilution plating on selective media. n = 4. Data are median from two independent experiments. (C) Growth kinetics in the presence of RegIIIβ. Mixture of STm strain SL1344 and E. coli strain LF82 was incubated with 10 µM RegIIIβ. Bacterial loads (CFU/ml) of individual strains were determined by selective plating: SL1344 was grown in agar medium containing streptomycin, whereas the agar medium containing ampicillin was used to isolate LF82. n indicates the number of data points. Bars, median with interquartile range. Unpaired t test. P > 0.05 not significant (ns), P < 0.05 (*), P < 0.01 (**), P < 0.001 (***), P < 0.0001 (****). (TIF) [file ppat.1013665.s007.tif]

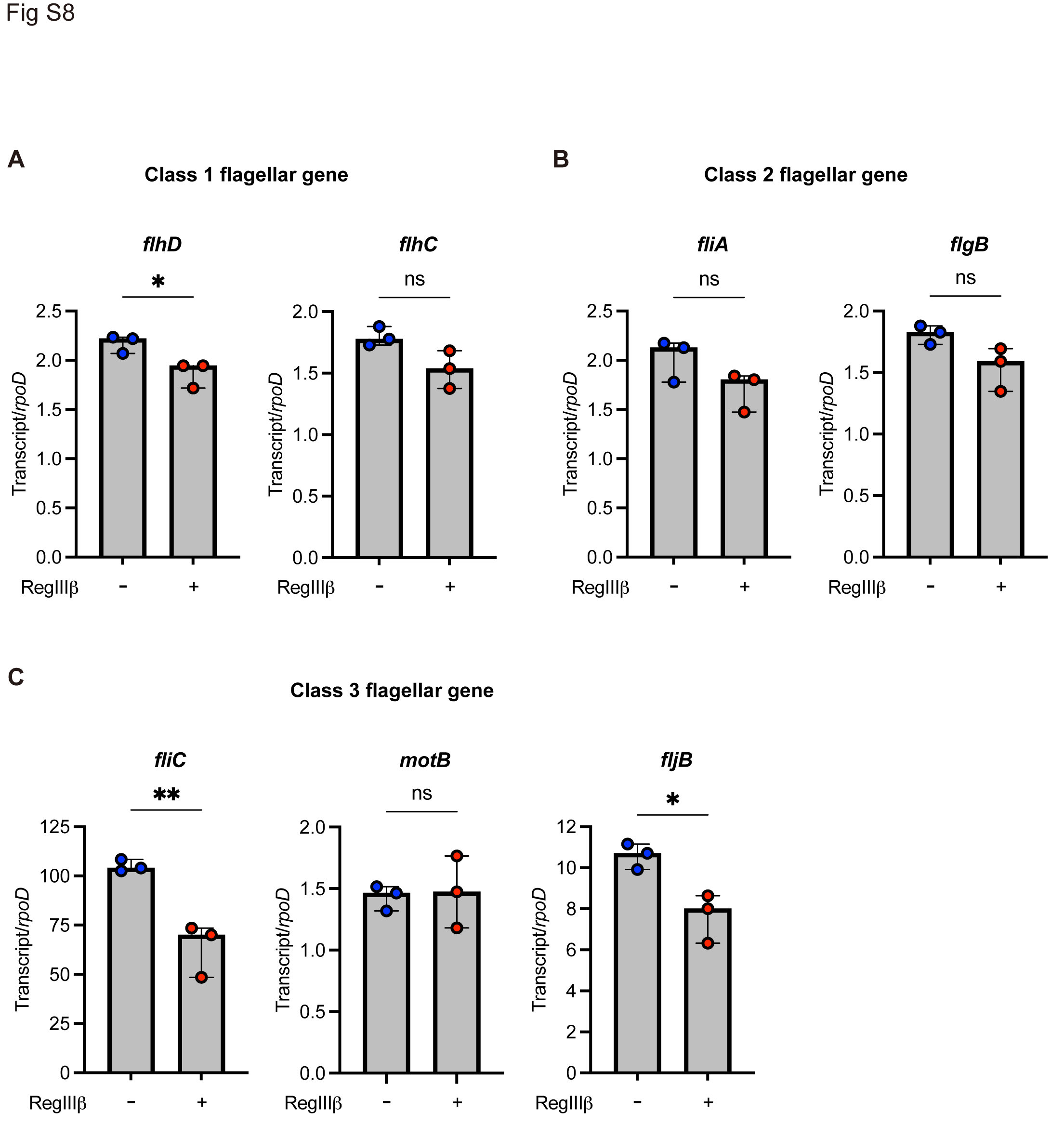

Supplement: S8 Fig — (A-C) Transcript levels of flagellar genes (class 1: flhD and flhC; class 2: fliA and flgB; class 3: fliC, motB and fljB) relative to rpoD. n is indicated by the number of dots. Bars, median with interquartile range. Unpaired t test. P > 0.05 not significant (ns), P < 0.05 (*), P < 0.01 (**), P < 0.001 (***), P < 0.0001 (****). (TIF) [file ppat.1013665.s008.tif]

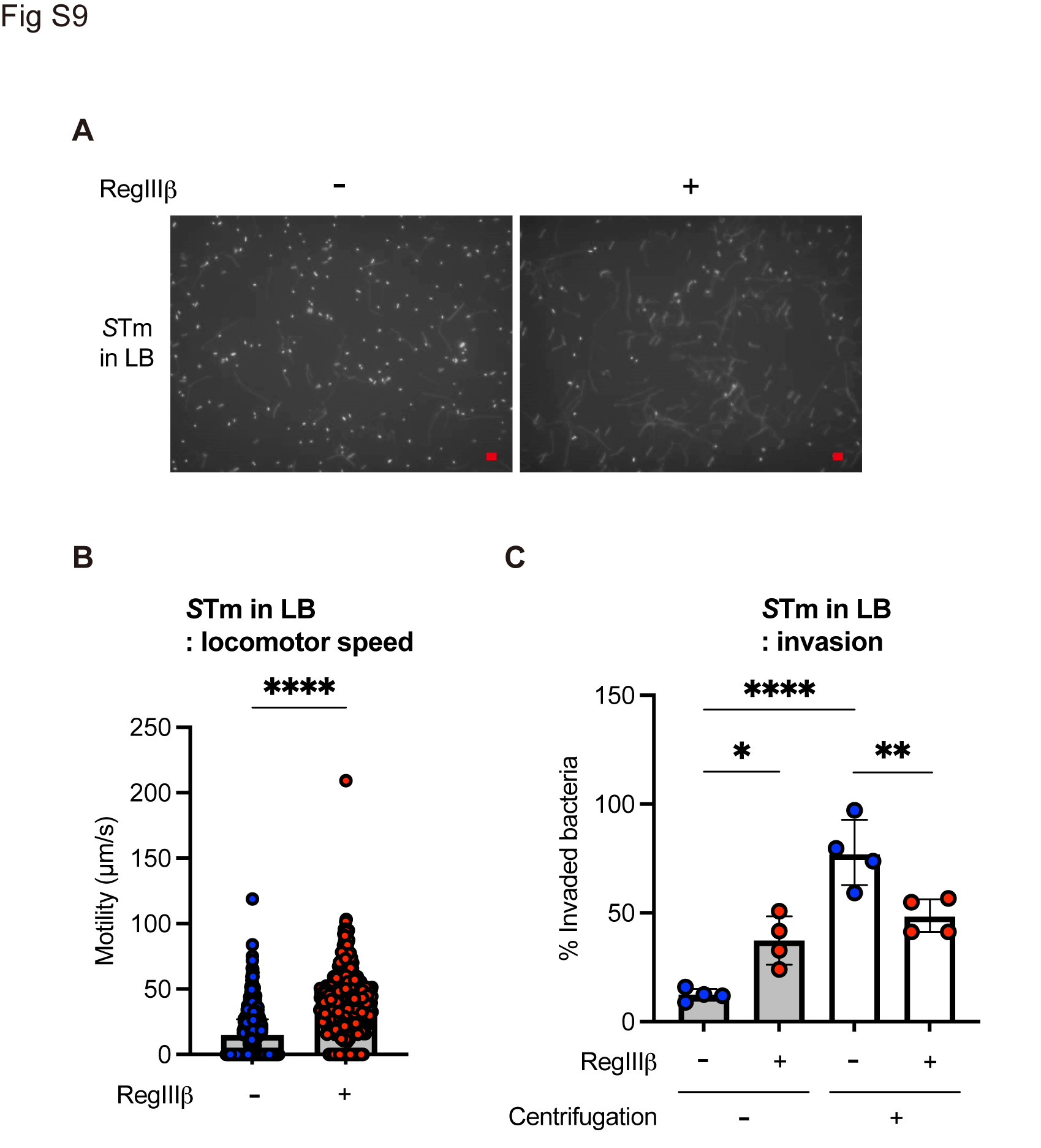

Supplement: S9 Fig — (A) Microscopy images of STm expressing green fluorescent proteins in LB medium. The bacterial strains were preincubated with recombinant RegIIIβ, placed on a glass slide, sealed under a glass coverslip, and observed by fluorescence microscopy (exposure time: 2.6 s). Red scale bar, 10 µm. (B) Microscopy quantification of STm locomotor velocity of the experiment in panel B. n = 808 [RegIIIβ-], and 556 [RegIIIβ+]. Bars, median with interquartile range. Two-tailed Mann‒Whitney U test. P > 0.05 not significant (ns), P < 0.05 (*), P < 0.01 (**), P < 0.001 (***), P < 0.0001 (****). (C) STm WT was preincubated with recombinant RegIIIβ in LB medium and added the monolayer cultures of HeLa cells, followed by centrifugation for close contact between STm cells and HeLa cells. Quantified invasiveness was determined by defining the input STm cells (inoculum) as 100%. n indicates the number of data points. Data were obtained from two independent experiments. Bars, median with interquartile range. A one-way ANOVA followed by Dunnett’s multiple comparisons test. P > 0.05 not significant (ns), P < 0.05 (*), P < 0.01 (**), P < 0.001 (***), P < 0.0001 (****). (TIF) [file ppat.1013665.s009.tif]

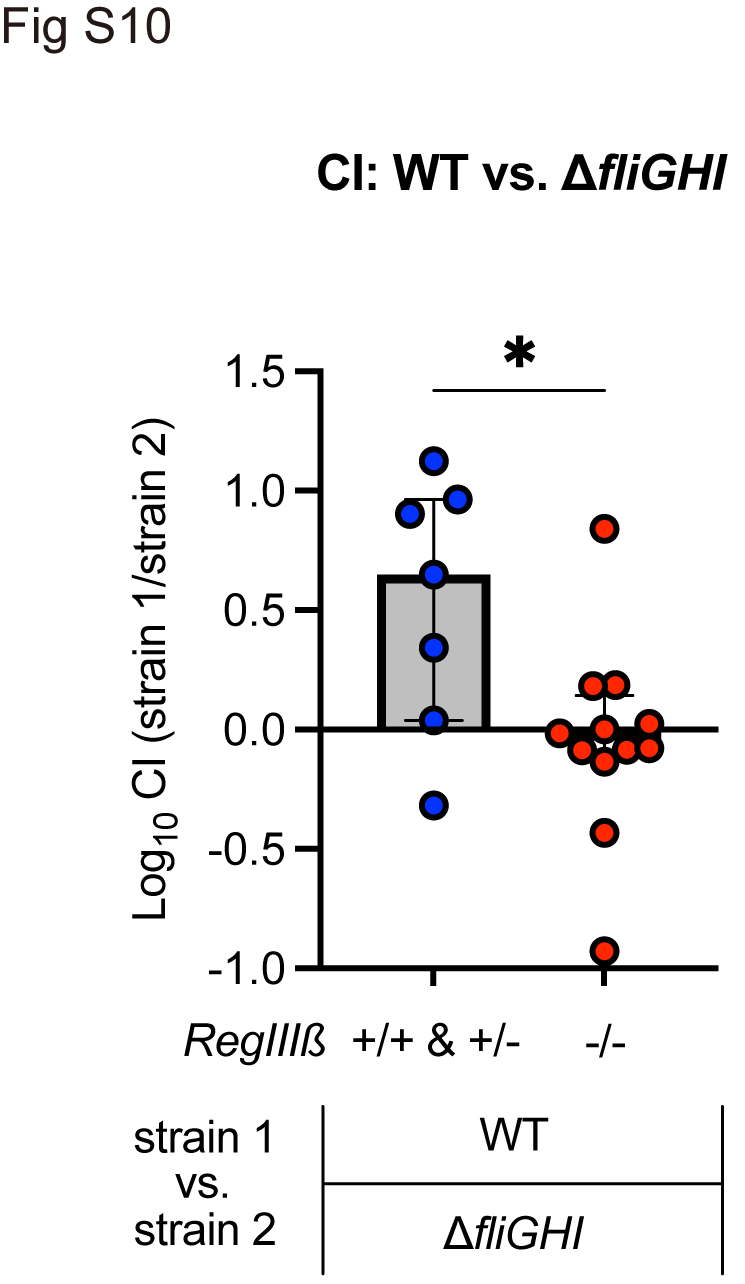

Supplement: S10 Fig — C57BL/6 mice were pre-treated with 25 mg of streptomycin by oral gavage 24 h before oral infection with STm (1:1 mixture of strain 1-WT and strain 2-∆fliGHI). Mice were euthanized on day 1 post-infection, and feces were collected. The CI of STm loads recovered from the feces was determined by selective plating. Bars, median with interquartile range. Two-tailed Mann‒Whitney U test. P > 0.05 not significant (ns), P < 0.05 (*), P < 0.01 (**), P < 0.001 (***), P < 0.0001 (****). (TIF) [file ppat.1013665.s010.tif]
