# Supplementary material for: RegIIIβ promotes Salmonella Typhimurium colonization of the gut in the early-stage gastrointestinal infection by enhancing flagella-driven locomotion
Source: PLoS Pathog. 2025 Nov 3;21(11):e1013665. doi: 10.1371/journal.ppat.1013665 (PMC12591440; doi:10.1371/journal.ppat.1013665)
Supplement: S1 Table — (DOCX) [file ppat.1013665.s011.docx]

**Table S1. Results of peptide matches using Mascot software**

| **Sample ID** | **Peptides** | **Identified protein** | **Predicted Mol. Mass (kDa)** |
| --- | --- | --- | --- |
| a | NPSTALDR | RegIIIβ | 19.5 |
|  | DMTCEVK |  |  |
| b | NPSTALDR | RegIIIβ | 19.5 |
|  | AFCGSLSR |  |  |
|  | WRDMTCEVK |  |  |
