# Supplementary material for: RegIIIβ promotes Salmonella Typhimurium colonization of the gut in the early-stage gastrointestinal infection by enhancing flagella-driven locomotion
Source: PLoS Pathog. 2025 Nov 3;21(11):e1013665. doi: 10.1371/journal.ppat.1013665 (PMC12591440; doi:10.1371/journal.ppat.1013665)
Supplement: S2 Table — (DOCX) [file ppat.1013665.s012.docx]

**Table S2. Bacterial strains and plasmids used in this study**

| **Strain or plasmid** | **Genotype** | **Reference** |
| --- | --- | --- |
| ***Salmonella enterica* serovar Typhimurium (*S*Tm)** | | |
| SL1344 | wild-type *S*Tm | [[1](#_ENREF_1)] |
| T330 | SL1344 harboring pACYC-gfp | [[2](#_ENREF_2)] |
| T273 | SL1344 ∆*fliGHI* | [[3](#_ENREF_3)] |
| T249  T523 | SL1344 ∆*invG* ∆*ssaV*::*cat*  SL1344 ∆*fliGHI::cat* | [[4](#_ENREF_4)]  [[3](#_ENREF_3)] |
| ***Escherichia coli* (*E*. *coli*)** | |  |
| mEC-1 | isolate from mouse | This study |
| LF82 | pathobiont isolated from an ileal biopsy sample of a patient with Crohn’s disease | [[5](#_ENREF_5)] |
| T650 | LF82 harboring pACYC-gfp | This study |
| **Plasmids** |  |  |
| pET11a-RegIIIβ | pET11a expressing RegIIIβ | [[6](#_ENREF_6)] |
| pET11a-RegIIIβ D142A | pET11a expressing point-mutated RegIIIβ D142A | [[7](#_ENREF_7)] |
| pACYC-gfp | pACYC184 expressing GFPmut3.1 | [[8](#_ENREF_8)] |

**Supplementary references**

1. Hoiseth SK, Stocker BA. Aromatic-dependent Salmonella typhimurium are non-virulent and effective as live vaccines. Nature. 1981;291(5812):238-9. doi: 10.1038/291238a0. PubMed PMID: 7015147.

2. Hoshino Y, Sakamoto T, Sudo N, Ito M, Haneda T, Okada N, et al. Fatty Acid Homeostasis Tunes Flagellar Motility by Activating Phase 2 Flagellin Expression, Contributing to Salmonella Gut Colonization. Infect Immun. 2022;90(7):e0018422. Epub 20220602. doi: 10.1128/iai.00184-22. PubMed PMID: 35652649; PubMed Central PMCID: PMCPMC9302153.

3. Nakamura N, Hoshino Y, Shiga T, Haneda T, Okada N, Miki T. A Peptidoglycan Amidase Activator Impacts Salmonella enterica Serovar Typhimurium Gut Infection. Infect Immun. 2020;88(6). Epub 20200520. doi: 10.1128/iai.00187-20. PubMed PMID: 32284369; PubMed Central PMCID: PMCPMC7240094.

4. Fujimoto M, Goto R, Haneda T, Okada N, Miki T. Salmonella enterica Serovar Typhimurium CpxRA Two-Component System Contributes to Gut Colonization in Salmonella-Induced Colitis. Infect Immun. 2018;86(7). Epub 20180621. doi: 10.1128/iai.00280-18. PubMed PMID: 29685984; PubMed Central PMCID: PMCPMC6013652.

5. Darfeuille-Michaud A, Neut C, Barnich N, Lederman E, Di Martino P, Desreumaux P, et al. Presence of adherent Escherichia coli strains in ileal mucosa of patients with Crohn's disease. Gastroenterology. 1998;115(6):1405-13. doi: 10.1016/s0016-5085(98)70019-8. PubMed PMID: 9834268.

6. Stelter C, Käppeli R, König C, Krah A, Hardt WD, Stecher B, et al. Salmonella-induced mucosal lectin RegIIIβ kills competing gut microbiota. PLoS One. 2011;6(6):e20749. Epub 20110609. doi: 10.1371/journal.pone.0020749. PubMed PMID: 21694778; PubMed Central PMCID: PMCPMC3111430.

7. Miki T, Holst O, Hardt WD. The bactericidal activity of the C-type lectin RegIIIβ against Gram-negative bacteria involves binding to lipid A. J Biol Chem. 2012;287(41):34844-55. Epub 20120815. doi: 10.1074/jbc.M112.399998. PubMed PMID: 22896700; PubMed Central PMCID: PMCPMC3464586.

8. Fujimoto M, Goto R, Hirota R, Ito M, Haneda T, Okada N, et al. Tat-exported peptidoglycan amidase-dependent cell division contributes to Salmonella Typhimurium fitness in the inflamed gut. PLoS Pathog. 2018;14(10):e1007391. Epub 20181031. doi: 10.1371/journal.ppat.1007391. PubMed PMID: 30379938; PubMed Central PMCID: PMCPMC6231687.
