# Supplementary material for: RegIIIβ promotes Salmonella Typhimurium colonization of the gut in the early-stage gastrointestinal infection by enhancing flagella-driven locomotion
Source: PLoS Pathog. 2025 Nov 3;21(11):e1013665. doi: 10.1371/journal.ppat.1013665 (PMC12591440; doi:10.1371/journal.ppat.1013665)
Supplement: S3 Table — (DOCX) [file ppat.1013665.s013.docx]

**Table S3. Oligonucleotide primers in this study**

| **Name** | **Sequence (5' to 3')** |
| --- | --- |
| qflhD-FW | CATATTTACTCCTTGCACAGCGTTT |
| qflhD-RV | GGTTCGTCTCCGCCAGTTT |
| qflhC-FW | GCTCGTCTACAAATGCTGGAAA |
| qflhC-RV | TGCTCCCAGGTCATAAACCA |
| qfliA-FW | GGCGTAATGCGACGGAAA |
| qfliA-RV | AGAGAAAAGTTGGCTGTTGTTGG |
| qflgB-FW | TTCAGCAGGAAGCGCTAAATC |
| qflgB-RV | GTCCACGCACCATCACCTT |
| qfliC-FW | CCGAAGTTGTTTCTATTGGTGGT |
| qfliC-RV | AGCGGGTTTTCGGTGGT |
| qmotB-FW | AAACAAACAGGCGGAACAGG |
| qmotB-RV | CGCTTTTGGCGATGTGG |
| SL qfljB-FW | GCTACGGGTGGTACGAATGG |
| SL qfljB-RV | CGGCATCAGCACCAGTAAAG |
