# Supplementary material for: RegIIIβ promotes Salmonella Typhimurium colonization of the gut in the early-stage gastrointestinal infection by enhancing flagella-driven locomotion
Source: PLoS Pathog. 2025 Nov 3;21(11):e1013665. doi: 10.1371/journal.ppat.1013665 (PMC12591440; doi:10.1371/journal.ppat.1013665)

Corresponding to Figure 1B

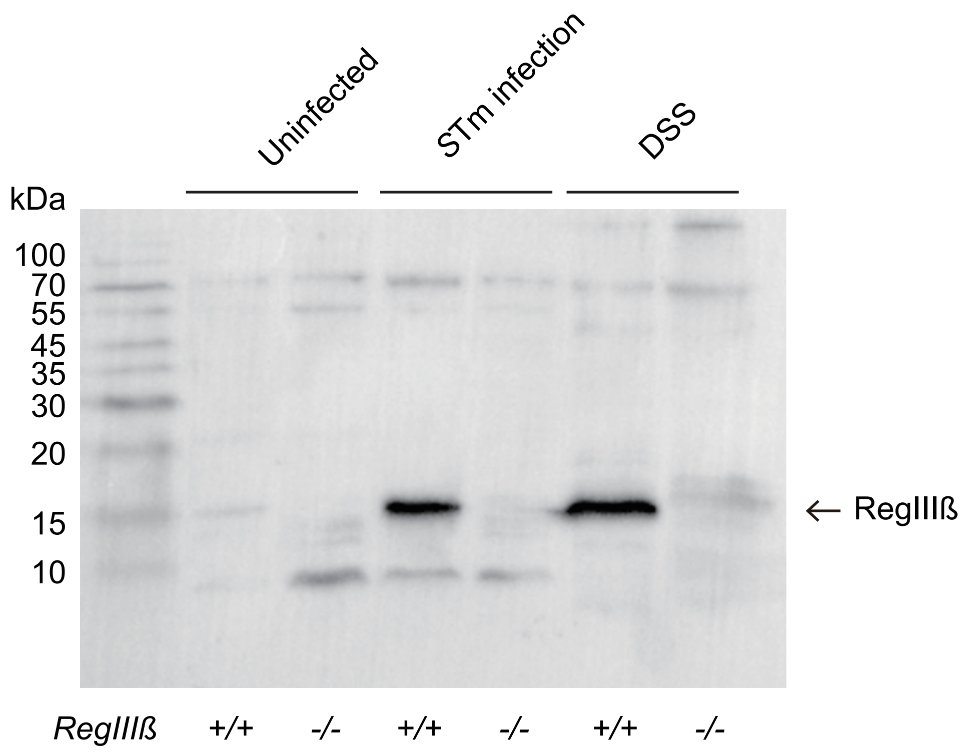

Corresponding to Figure 1F

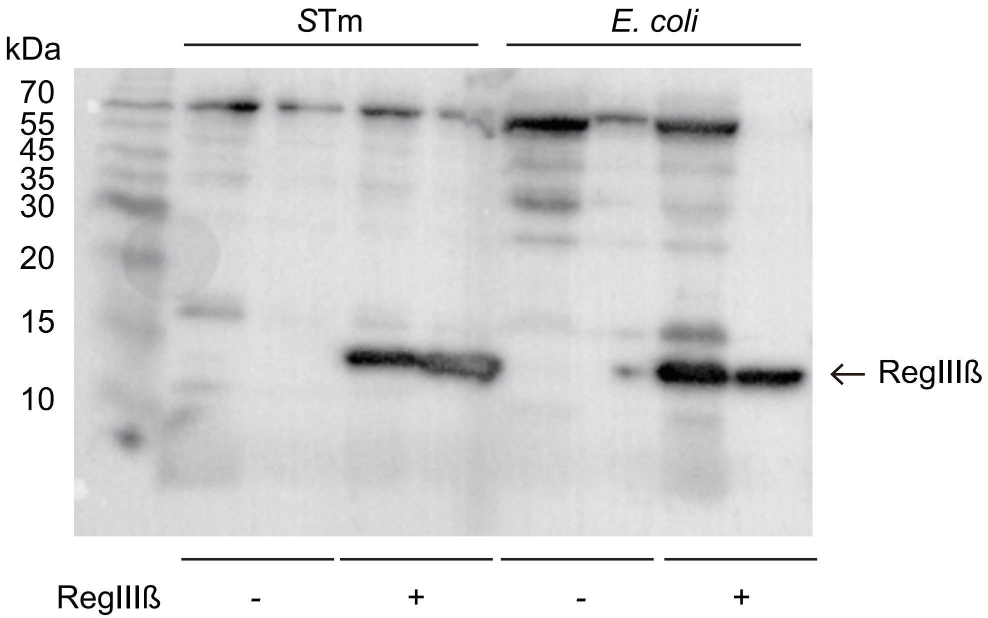

Corresponding to Figure 2E

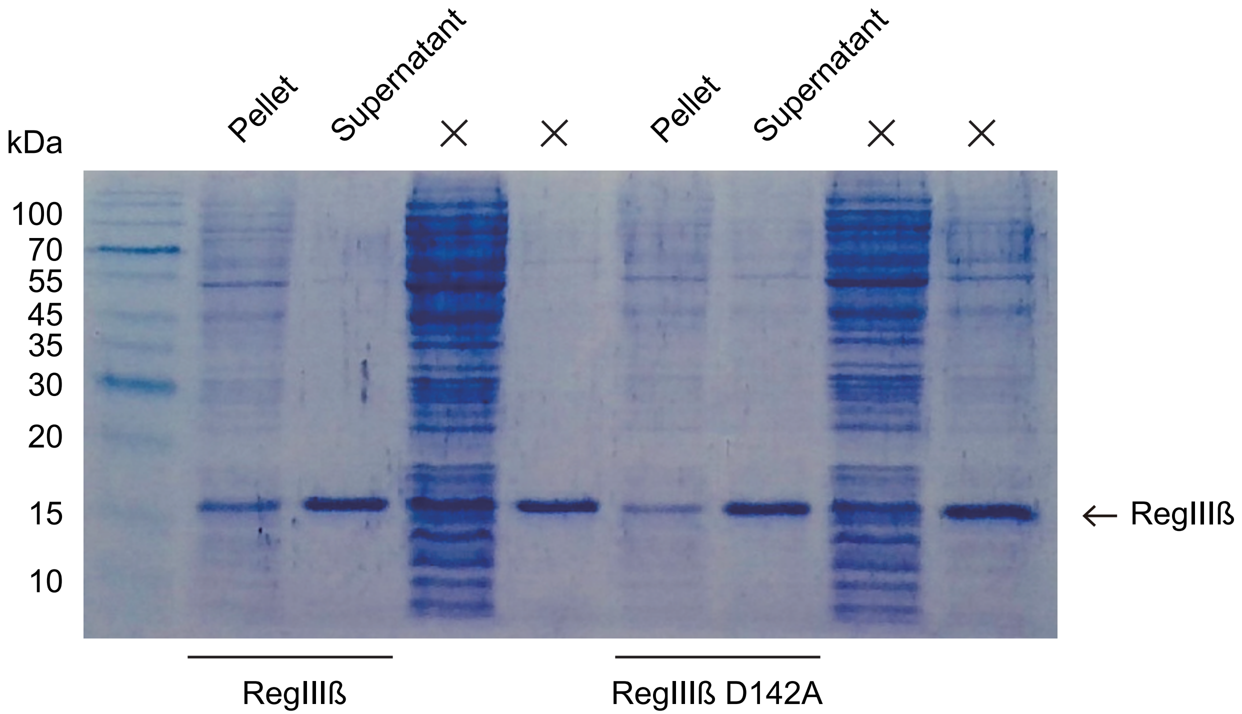

Corresponding to Figure 3A

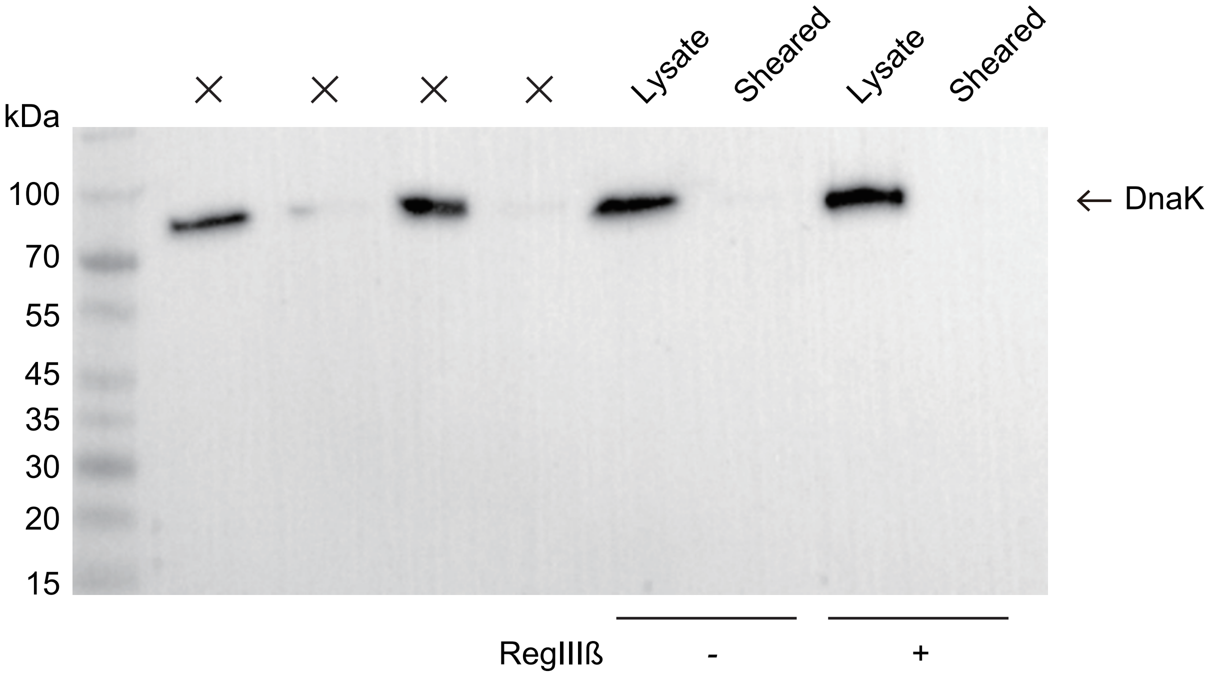

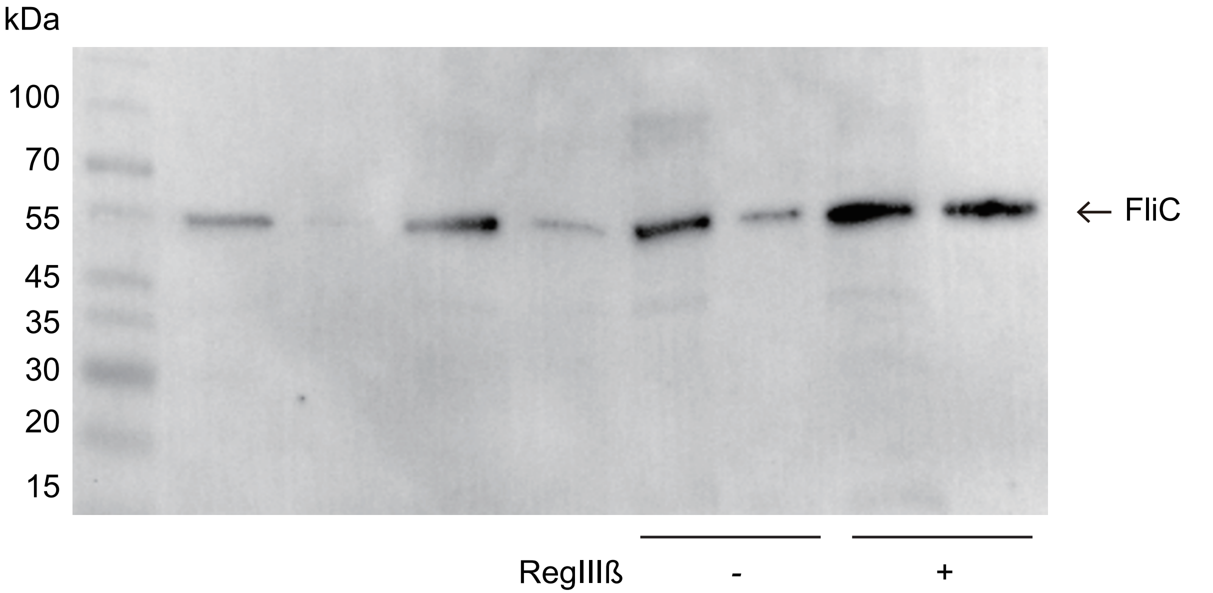

Corresponding to Figure S1A

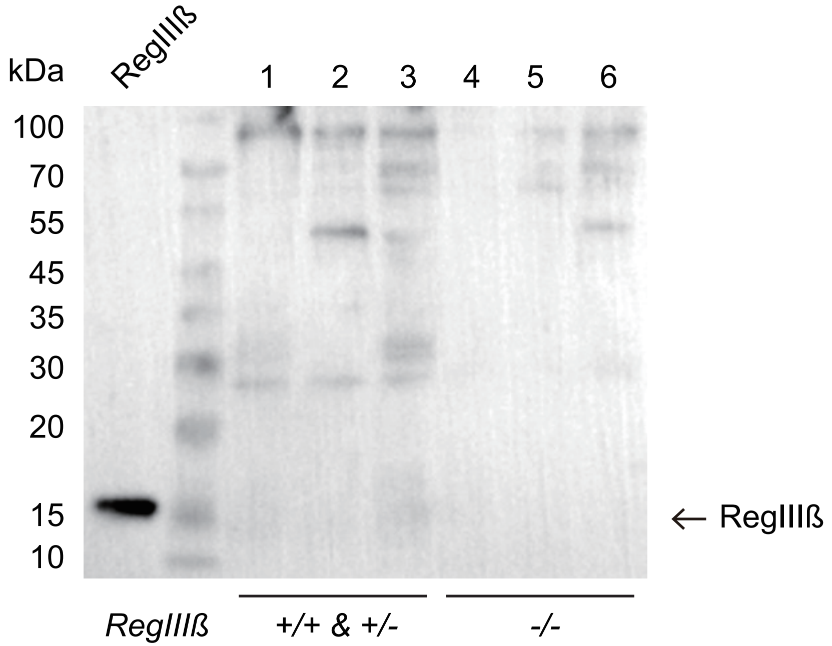

Supplement: S1 Raw Images — (PDF) [file ppat.1013665.s015.pdf]
